# Habitat-Driven Insect Community Structure in an Arid Agroecosystem of Xinjiang, China: Integrated Light-Trap Monitoring and DNA Barcoding Evidence

**DOI:** 10.3390/insects17080777

**Published:** 2026-07-28

**Authors:** Muhammad Irfan Zafar, Shaoshan Wang, Qizhi Liu, Jawad Hassan, Talha Shafique, Yuxian Liu

**Affiliations:** 1 Xinjiang Production and Construction Corps Key Laboratory of Oasis Agricultural Pest Management and Plant Protection Resources Utilization, College of Agriculture, Shihezi University, Shihezi 832003, China; irfanzafar595@gmail.com (M.I.Z.); lqzzyx163@163.com (Q.L.); lyx18595671589@163.com (Y.L.); 2 Laboratory of Entomology and Nematology, College of Plant Protection, China Agricultural University, Beijing 100193, China; 3 Institute of Biodiversity, School of Ecology and Environmental Science, Yunnan University, Kunming 650500, China; j.nasir15786@gmail.com; 4 Department of Knowledge Research Support Service (KRSS), University of Management and Technology, Lahore 54700, Punjab, Pakistan; talhashafique300@gmail.com

**Keywords:** DNA barcoding, insect biodiversity, pest monitoring, light trap, novel species

## Abstract

Research on insect biodiversity in arid and semi-arid agroecosystems of Central Asia remains limited, hindering the development of effective insect conservation strategies and integrated pest management (IPM) programs. This study examined insect community structure, diversity, and seasonal dynamics across three habitat types in Kekedala City, in the autonomous region of Ili Kazakh, Xinjiang, China. Solar-powered ultraviolet LED light traps (350–420 nm) were used for sampling from April to August 2024. A total of 63,090 individuals of 83 species, 57 families and 72 genera of insects were collected. Diversity indices calculated using the complete species dataset showed highest diversity in the riparian corridor (L3: *H′* = 3.42, *D* = 0.962, *S* = 62), intermediate diversity in the agro-horticultural garden (L2: *H′* = 3.18, *D* = 0.948, *S* = 54), and lowest diversity in the xerophytic shrubland (L1: *H′* = 2.97, *D* = 0.935, *S* = 47). A supplementary analysis targeted a shared core assemblage of 17 species, defined as taxa captured at all three sampling sites with cumulative total abundances ranging from 50 to 100 individuals. This subset exhibited near-uniform diversity metrics across habitats (*H′* = 2.83, *D* = 0.941, Pielou’s evenness J′ ≈ 1.0), a pattern driven by the prevalence of habitat generalists rather than capturing site-specific full community diversity. Two abundant pest species, *Helicoverpa armigera* (Hübner, 1808) (cotton bollworm, 5209 individuals, 8.3% of total) and *Oryctes rhinoceros* (Linnaeus, 1758) (rhinoceros beetle, 5206 individuals, 8.2% of total), both peaked in abundance in July–August; *O. rhinoceros* is associated with local ornamental and date palm plantings in the study area. In total, 15 major pest species were recorded. Mitochondrial cytochrome c oxidase subunit I (COI) DNA barcoding confirmed two new national insect records for China: *Stethoconus pyri* (Mella, 1861) (Hemiptera: Anthocoridae) and *Pinacoplus didymogramma* (Hampson, 1907) (Lepidoptera: Noctuidae). This study provides the first comprehensive baseline inventory of the insect fauna of Kekedala City. Descriptive ordination analyses suggest that habitat vegetation complexity may exert a stronger filtering effect on insect assemblage composition than the relatively small seasonal variation in temperature and humidity observed during the sampling period. As only a single trap was deployed within each habitat with no within-site replication, all cross-habitat community patterns reported herein are strictly descriptive and require validation via replicated field sampling in future research.

## Simple Summary

Insect communities across arid agricultural landscapes in Central Asia remain severely neglected, hindering effective crop pest control and wildlife conservation strategies. This research surveyed insect biodiversity across three distinct natural and agricultural landscapes in Kekedala City, Xinjiang, China: drought-resistant xerophytic shrubland, a horticultural garden, and a riparian vegetation corridor. Solar-powered ultraviolet light traps sampled nocturnal insects April–August 2024, collecting 63,090 individuals from 83 species. Riparian corridors held the highest insect diversity, followed by the horticultural garden, while xerophytic shrubland supported the lowest. Two dominant pests, cotton bollworms (*Helicoverpa armigera*) and rhinoceros beetles (*Oryctes rhinoceros*), peaked in July and August, with 15 total damaging crop species recorded. COI DNA barcoding uncovered two *Stethoconus pyri* (Mella, 1861) and *Pinacoplus didymogramma* (Hampson, 1907) insect species newly documented for China, confirming Xinjiang as a biogeographic transition zone linking Central and East Asian insect faunas. Habitat vegetation complexity structured insect assemblages more strongly than minor seasonal temperature and humidity fluctuations. This study delivers Kekedala’s first comprehensive insect baseline research, yet single unreplicated traps prevent formal statistical verification. Offsetting seasonal activity of pests and beneficial insects requires continuous integrated pest management, and this dataset enables long-term biodiversity tracking, pest outbreak prediction and habitat conservation amid agricultural intensification and climate change.

## 1. Introduction

Insects comprise more than half of all described species on Earth and perform essential ecological functions, including pollination, decomposition, nutrient cycling, biological control, and energy flow across trophic levels [1]. Insects respond rapidly to changes in vegetation structure, temperature, relative humidity, and land use, making them sensitive bioindicators of ecosystem health. Insect populations are undergoing widespread declines globally, highlighting the need for baseline biodiversity surveys, especially in understudied regions experiencing rapid agricultural expansion and habitat transformation [2,3,4]. Arid and semi-arid regions cover approximately one-third of Earth’s land surface yet remain among the least-studied environments for insect biodiversity and Xinjiang Uyghur Autonomous Region in northwestern China is one such region [5,6,7]. Kekedala City, located in the Ili Kazakh Autonomous Prefecture, is an important agricultural region where cotton and maize are the dominant crops [8,9,10].

Economically important insect pests, including cotton bollworms, scarab beetles and aphids, cause substantial annual crop losses across the region. Identifying insect species, their seasonal activity, and their natural enemies is an essential component of pest management and no prior baseline biodiversity assessments integrating continuous light-trap surveillance and COI DNA barcoding have been implemented in Kekedala’s mixed cotton–maize–orchard agroecosystem. Regional entomological research to date has focused narrowly on pest assemblages associated with single crop types, with almost no comparative analyses of insect communities across divergent habitat classes [11,12,13]. Reliable baseline data on insect biodiversity across multiple habitats are therefore urgently needed to support targeted regional pest mitigation, yet such integrated datasets remain unavailable for Kekedala’s unique arid agricultural landscape. This study deployed automated solar UV LED light traps alongside COI-based DNA barcoding to confirm uncertain morphological identifications of cryptic, poorly documented regional insect taxa for which regional identification keys are unavailable. Light traps are effective for capturing nocturnal insects in a consistent manner. Data collected throughout the trapping period can be related to environmental conditions and crop growth stages, providing valuable information for predicting insect activity and identifying vulnerable stages for pest management [14,15,16].

This study aimed to document insect diversity across three habitats within the city, quantify spatial and temporal variation in diversity, evaluate the influence of temperature, humidity and habitat type on insect biodiversity and identify species newly recorded in China through DNA barcoding. Previous studies have provided limited information on the influence of habitat type on insect biodiversity. The present study demonstrates that riparian vegetation corridors support greater insect diversity than adjacent xerophytic shrublands and managed agro-horticultural habitats. This study represents the first comprehensive survey of insect biodiversity conducted in this arid and semi-arid zone of Kekedala city that provides a foundation for optimizing pest management in Xinjiang cotton, maize and horticultural crops (apple, apricot, plum and ornamental plants). We formalized two primary a priori hypotheses: (1) riparian corridors support higher insect species richness, elevated community diversity, and taxonomically distinct insect assemblages relative to xerophytic shrublands and managed agro-horticultural gardens; (2) fine-scale seasonal shifts in temperature and relative humidity exert weaker filtering effects on insect community composition than habitat vegetation structural complexity.

## 2. Materials and Methods

### 2.1. Study Area

Insect sampling was conducted at three sites representing distinct habitat types in the Kekedala region (Ili Kazakh Autonomous Prefecture, Xinjiang, China; ~43.3° N, 80.5° E), located in the Ili River valley at approximately 540–560 m elevation. The region has a temperate arid continental climate, with hot dry summers and cold winters. The three sites were separated by 2.5–4.2 km to maximize spatial independence, and each site contained one solar-powered UV light trap: Site Location 1 (L1)—Xerophytic shrubland (43.312° N, 80.487° E, 562 m a.s.l.): A semi-natural, undisturbed shrub-dominated habitat with sparse vegetation cover (~35%). Dominant plant species include *Haloxylon ammodendron*, *Tamarix ramosissima*, *Reaumuria soongorica*, and *Nitraria tangutorum*. The trap was installed 1.5 m above ground on a metal pole in an open patch with unobstructed exposure, >50 m from the nearest habitat edge, while no *Arecaceae* (palm) host vegetation occurs within the L1 shrubland boundary; all ornamental date palm plantations are situated 3–6 km away in regional urban and oasis horticultural zones. Site Location 2 (L2)—Agro-horticultural garden (43.298° N, 80.521° E, 548 m a.s.l.): A managed horticultural site with mixed fruit trees (apple, pear, apricot, and walnut), ornamental shrubs, and herbaceous ground cover (~70% total vegetation cover). The site is maintained with regular irrigation and minimal pesticide use. The trap was placed 1.5 m high at the garden edge, adjacent to a field boundary with natural vegetation. Site Location 3 (L3)—Riparian corridor (43.305° N, 80.543° E, 541 m a.s.l.): A wetland corridor along a seasonal tributary of the Ili River, with dense *Populus alba* and *Salix babylonica* trees, a lush herbaceous understory, and high vegetation cover (~90%) as shown below Table 1. 

**Figure 1:**
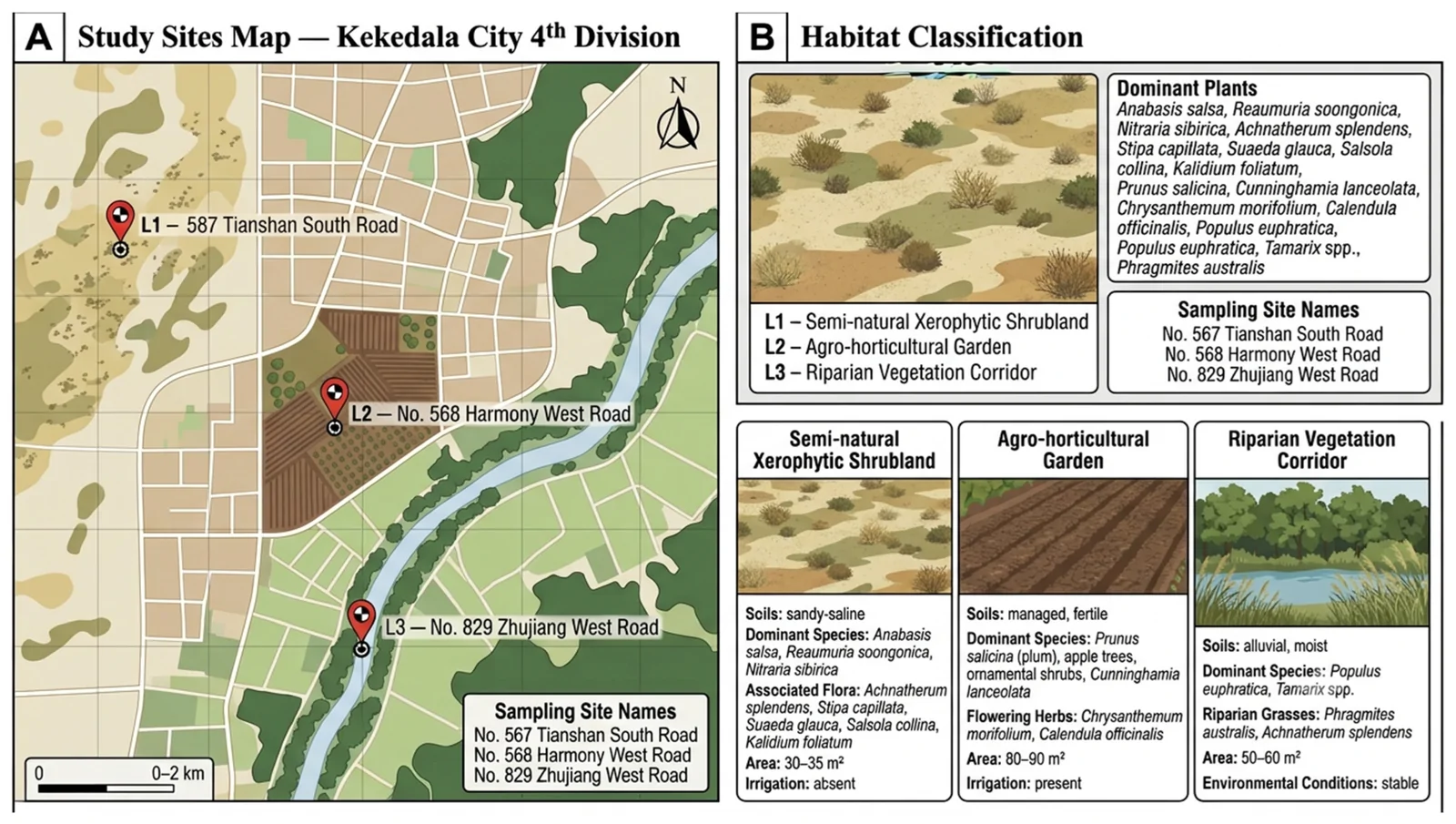
(**A**) Map of the study area in Kekedala, Xinjiang, China, showing the locations of the three light trap sites: L1 (xerophytic shrubland), L2 (agro-horticultural garden), and L3 (riparian corridor). (**B**) shows the habitat classification and key characteristics of each site type.

The trap was installed 1.5 m above ground in a forest gap approximately 20 m from the stream bank. All traps operated continuously from 1 April to 31 August 2024, with insect collections made every 7 days. The habitat characteristics and spatial distribution of the three selected study sites within the 4th Division of Kekedala City are presented in Figure 1.

#### Sampling Design

Field implementation of this baseline insect biodiversity survey was constrained by limited project funding and limited timeline; consequently, a single solar-powered UV light trap was installed within each distinct habitat type. This sampling design did not include within-habitat replication at each sampling location. All inter-habitat community comparisons presented throughout this manuscript are therefore strictly descriptive rather than inferential; formal multivariate statistical testing (e.g., PERMANOVA) is not feasible for this dataset, as such analyses require replicated sampling units to compute residual degrees of freedom [17,18]. Future studies incorporating replicated sampling are required to statistically validate these observational patterns. The primary objective of this single-trap sampling framework was to generate the first comprehensive species inventory for Kekedala’s arid agricultural landscape.

### 2.2. Light Trap Design and Deployment

**Figure 2:**
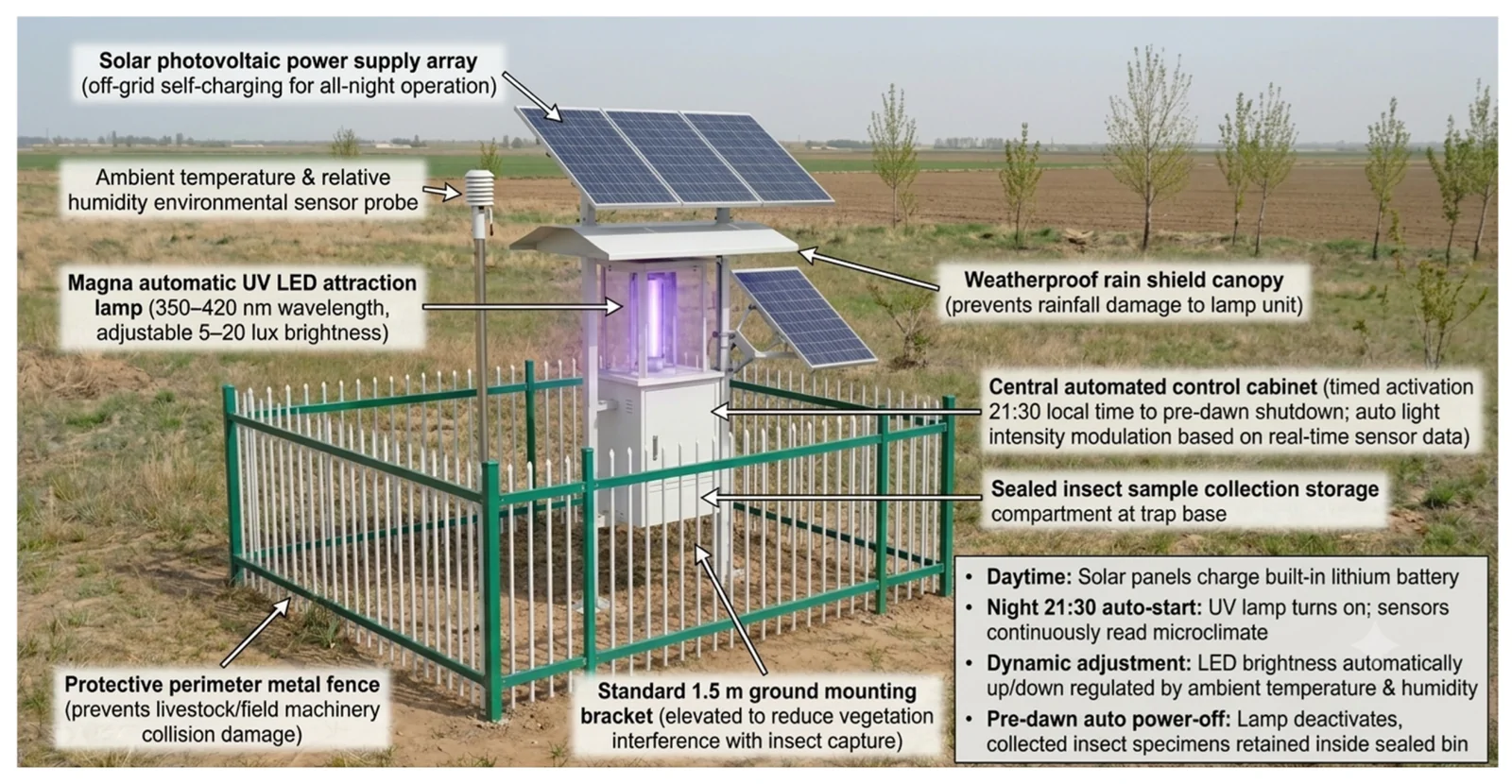
Solar-powered automatic UV LED insect monitoring trap deployed across three arid agricultural habitats in Kekedala City, Xinjiang, China. The annotated schematic is built upon original in situ field photographs of the physical sampling trap. Key functional modules are labelled: solar photovoltaic power supply array, temperature/humidity environmental sensor probe, adjustable-wavelength UV LED attraction lamp, weatherproof rain shield canopy, automated central control cabinet, sealed insect specimen storage compartment, 1.5 m elevated mounting bracket, and livestock-protective perimeter metal fence. A summary of automated day-night operational logic is provided in the bottom-right text box to detail the trap’s timed sampling workflow. Disclosure statement for journal AI policy: Base visual material consists entirely of original field photographs captured at sampling sites; artificial intelligence graphic tools were only used for post-processing to organize annotation arrows, standardize text boxes, and improve visual clarity. No fully AI-generated synthetic trap imagery was created, and all hardware specifications match the physical field equipment.

Solar-powered automated UV LED light traps (Magna model, Russell IPM, Hawarden, UK) were deployed for insect sampling, emitting light at a wavelength range of 350–420 nm with adjustable brightness of 5–20 lux. At each sampling location, traps were mounted 1.5 m above ground within open vegetation gaps to minimise obstructive shading from surrounding plant biomass [19,20]. Each trap activated automatically at 21:30 local time and ceased operation before dawn; integrated environmental sensors dynamically adjusted LED light intensity in response to ambient temperature and relative humidity fluctuations. This approach is commonly used in baseline biodiversity surveys conducted under comparable field conditions; a single trap per habitat, however, does not provide any replication within each habitat [17,18]. For future studies, at least 3 independent traps should be used per habitat in order to be able to properly test for statistics. Photographs of the light trap design, structural components, and field installation used for insect monitoring are shown in Figure 2. The system consisted of a solar-powered automated light trap equipped with illumination and insect attraction components, installed under open-field conditions across the sampling locations. The trap operated continuously throughout the monitoring period and maintained stable performance under varying environmental conditions. A fully labelled figure of the UV light trap is provided to standardize trap deployment protocols for future regional replicated monitoring surveys. All traps were mounted 1.5 m above ground in open, unobstructed gaps with >50 m buffer distance from large tree canopies or tall dense shrubs to avoid blocking UV light emission and reduce uneven insect attraction. Livestock exclusion fencing around each trap base prevented physical damage to sampling hardware and disturbance of captured insect material.

### 2.3. Sampling Protocol and Taxonomic Identification

Daily insect collections were performed between 07:30 a.m. and 09:30 a.m. local time, with specimens sorted by habitat site (L1, L2, L3) immediately post-retrieval. Bulk insect material was transported to the laboratory within insulated coolers and subsequently frozen at −20 °C for a minimum of four hours to humanely euthanize individuals while preserving intact morphological diagnostic traits. Post-freezing, intact individuals were pinned, wing spread for Lepidoptera and large Coleoptera, and labelled including sampling date, habitat site code (L1/L2/L3). The identification was conducted using regional and global insect morphological taxonomic keys [21,22]. Morphological identifications relied on published regional insect taxonomic keys for Xinjiang agroecosystems and global family-level diagnostic guides for Lepidoptera, Coleoptera, Hemiptera, Diptera and Hymenoptera. Specimens with ambiguous diagnostic traits were sent to specialist entomologists for morphological validation prior to DNA barcoding screening (for new recorded species only). When morphological identification was uncertain, expert taxonomists were consulted. The collected specimens are permanently deposited in the entomological collection of the Department of Plant Protection, College of Agriculture, Shihezi University, Xinjiang, China. Voucher specimen numbers will be assigned during museum cataloguing and will be provided after accession into the institutional collection. All 83 species are listed, with their order and family, total numbers and distribution across sites in Supplementary File S1.

### 2.4. Species Inclusion Criteria for Diversity Index Calculations

All statistical analyses are descriptive, as the study uses one trap per habitat with no within-habitat replication. Formal statistical comparison of habitat-level means is not possible with this design. To characterize insect community diversity and structure, we calculated species richness (*S*), Shannon–Wiener diversity (*H′*), Simpson’s diversity (*D*), and Pielou’s evenness (*J′*) for each habitat. We also generated species accumulation curves and rarefaction curves to assess sampling completeness and compare richness standardized by sampling effort. Non-metric Multidimensional Scaling (NMDS) was used to visualize differences in community composition among the three habitats based on Bray–Curtis dissimilarity, for descriptive purposes only [23,24,25].

As a supplementary analysis, we also calculated diversity indices for a defined core assemblage of 17 species. This subset comprised species that satisfied two fixed, pre-defined inclusion criteria: (1) recorded at all three sampling habitats (L1, L2, L3), and (2) cumulative total abundance range with >50 to 100 individuals across the full April–August sampling window. The threshold of >50 to 100 individuals was selected to filter rare transient species and retain only widespread, abundant taxa for comparative core community analysis. This core assemblage analysis was used to compare the structure of the shared, abundant component of the community across habitats and to reduce noise from rare, transient species. We emphasize that this analysis does not represent total site diversity, which is presented as the primary result using the full species dataset.

### 2.5. Environmental Data

Calibrated data loggers at the height of the traps were used to record mean daily air temperature (°C) and relative humidity (RH) (%) at each trap site. These records were compared with the data on insect abundance that were recorded every day to assess the relationship between environmental conditions and insect abundance [26,27]. Environmental data loggers recorded daily mean temperatures ranging from 12.4 °C to 31.8 °C and mean relative humidity ranging from 28.3% to 62.1% across the April–August sampling period, consistent with the continental arid climate of the region.

### 2.6. Extraction of DNA and COI Barcoding

Two morphologically ambiguous specimens were selected for molecular identification, as critical diagnostic traits including wing venation patterns, antennal segmentation, and head capsule morphology failed to match descriptions within available regional taxonomic identification keys. Leg and thoracic tissues were homogenized using a MM400 ball mill (Retsch GmbH, Haan, Germany) at 15 r/s for 2 min with 30–90 mg of each tissue [28,29]. The genomic DNA was isolated using a magnetic bead kit for animal tissue (GenMagBio, Beijing, China). DNA quality was assessed using a Nanodrop 500 microvolume spectrophotometer (Allsheng Instruments Co., Ltd., Hangzhou, Zhejiang, China), yielding A260/A280 values of 2.10 and 2.14, respectively, and concentrations of 1401.4 ng/µL and 22.1 ng/µL, respectively [30,31,32]. The large difference in extracted DNA concentrations stems from pronounced interspecific differences in body size and usable tissue biomass: *Pinacoplus didymogramma* (Hampson, 1907) is a large noctuid moth, whereas *Stethoconus pyri* (Mella, 1861) is a minute anthocorid heteropteran [25]. Despite the difference in DNA concentration, both samples had high purity (A260/A280 = 2.10 and 2.14) and produced high-quality sequences, with the lower concentration sample compensated by using increased template volume in PCR.

The 658 bp COI barcode fragment was amplified in a total 25 µL PCR reaction system consisting of 12.5 µL 2× Rapid Taq Master Mix (Vazyme, Nanjing, China), 2.0 µL each of forward primer LEP-F1 (5′-ATTCAACCAATCATAAAGATATTGG-3′) and reverse primer LEP-R1 (5′-TAAACTTCTGGATGTCCAAAAAATCA-3′), 2.0 µL template DNA, and 6.5 µL nuclease-free ddH_2_O. Thermal cycling parameters were as follows: initial denaturation at 94 °C for 4 min; 35 iterative cycles of 94 °C denaturation (30 s), 52 °C annealing (45 s), and 72 °C extension (1 min); followed by a terminal elongation step at 72 °C for 10 min. The PCR products were analysed on a 1.5% agarose gel, purified and then sequenced in both directions by Shanghai Bioengineering Co., Ltd. Shanghai, Chine. Sequences were assembled and edited in MEGA 6.06 and compared with sequences retrieved from GenBank using BLASTn (version 2.14.1) and with BOLD Systems v4.0.

### 2.7. Statistical Analysis

Primary diversity analysis was conducted using the complete species dataset for each habitat to accurately represent total community diversity. We calculated four standard diversity metrics: observed species richness (*S*), Shannon–Wiener diversity index (*H′*), Simpson’s diversity index (*D*), and Pielou’s evenness index (*J′*).

As a secondary, supplementary analysis, we also calculated diversity indices for a defined “core assemblage” of 17 species. This subset includes only species that met two objective criteria: (1) present at all three study sites, and (2) with >100 total individuals across the sampling period. This core assemblage analysis was used to compare the structure of the shared, abundant component of the community across habitats. We note that this filtered subset reduces among-habitat variation and does not represent total site diversity; it is presented only for comparative analysis of the dominant shared species pool.

Community composition was visualized using Non-metric Multidimensional Scaling (NMDS) ordination based on Bray–Curtis dissimilarity of log-transformed abundance data, for descriptive purposes only. Raw species abundance counts were log_10_(x + 1) transformed prior to Bray–Curtis dissimilarity calculation to reduce bias from hyper-abundant pest species (e.g., *Helicoverpa armigera*, *Oryctes rhinoceros*). Species rarefaction curves were standardised to the minimum total individual count recorded across all three habitats to enable unbiased richness comparison independent of sampling yield. Species accumulation curves and rarefaction curves were generated to assess sampling completeness. All analyses were performed in R v4.3.1 using the vegan package.

## 3. Results

### 3.1. Data on the Abundance and Taxonomic Composition of Insects in Detail

A total of 63,090 individuals of insects were collected over 5 months. These represented 83 species, 57 families and 72 genera (Supplementary File S1). Among the 83 recorded species, 17 occurred at all three study sites and met the predefined criteria for inclusion in the supplementary core-assemblage diversity analysis. The remaining 66 species occurred at low abundance or at only one or two sites. These species are included in Supplementary File S1, but not in the calculations of the diversity index.

Taxonomically, Lepidoptera and Coleoptera constituted the largest share of collected individuals and species, followed sequentially by Diptera, Hemiptera and Hymenoptera: *Cicindela ocellata* (Klug, 1834), *Paracle fusca* (Walker, 1856), *Helicoverpa armigera* (Hübner, 1808), *Oryctes rhinoceros* (Linnaeus, 1758) and *Polyphylla ragusae* (Reitter, 1897). Within the core shared species assemblage, *Protoschinia nuchalis* (Guenée, 1852) exhibited the lowest total capture count (4757 individuals). Rank–abundance curves revealed a steep drop in relative abundance among dominant taxa, followed by a gradual, steady decline across rarer species.

### 3.2. Family-Level Species Richness Across Study Sites

**Figure 3:**
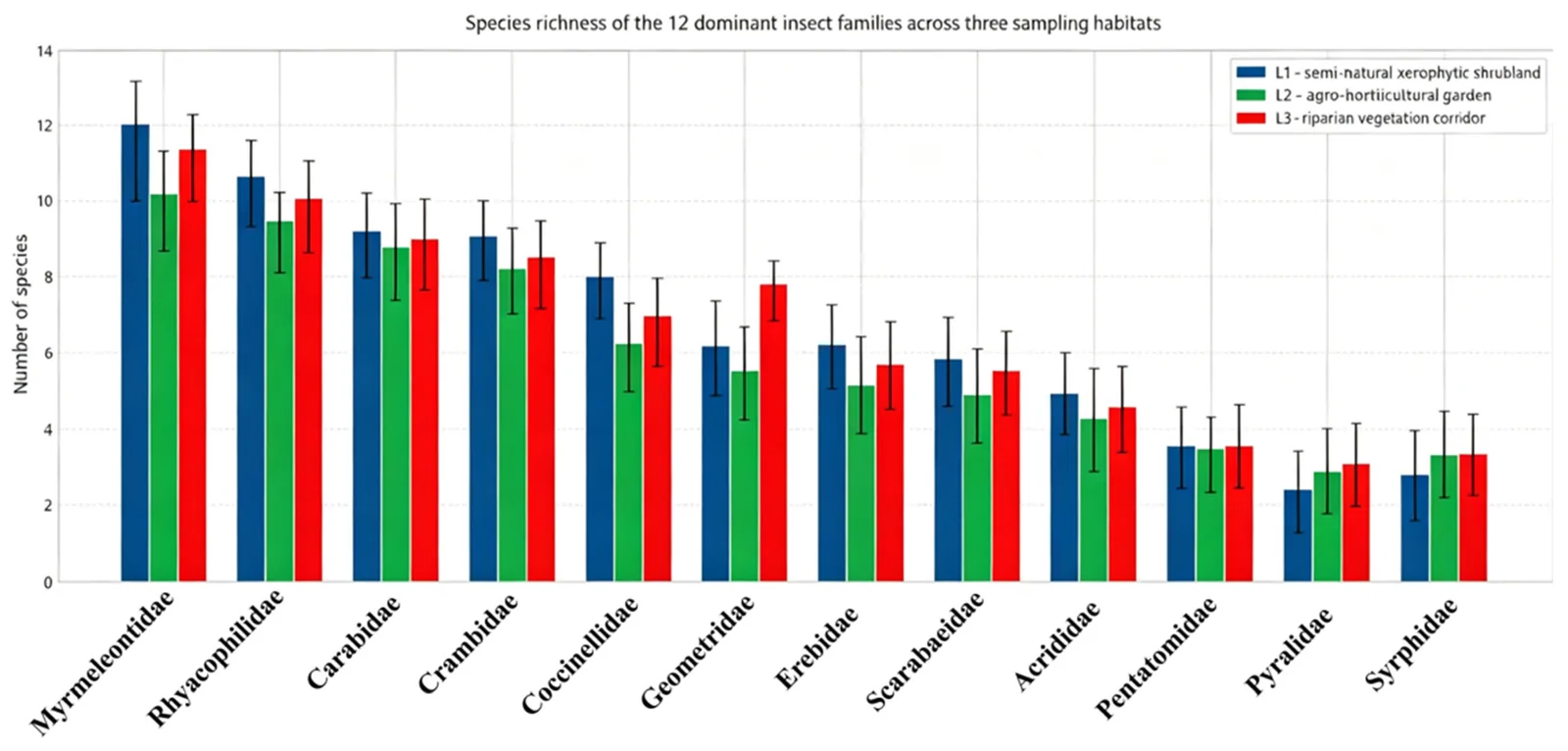
Species richness of the 12 most abundant insect families across three study sites in Kekedala City, Xinjiang, China. Bars represent number of species per family at each site. L1 = semi-natural xerophytic shrubland (dark blue); L2 = agro-horticultural garden (green); L3 = riparian vegetation corridor (red).

Species richness was highest at the family level, with 12 families dominating the species (Figure 3). The highest total species richness among the families in the entire study area was found in the family Myrmeleontidae (L1 = 22, L2 = 16, L3 = 15; total = 53). This pattern is consistent with the preference of these taxa for dry, open, sandy habitats. The richness of the Carabidae was higher at L1 (*n* = 10) than at L2 (*n* = 3) and L3 (*n* = 4), which is consistent with their preference for bare or lightly vegetated soils. The richness of Rhyacophilidae was relatively high in L1 (*n* = 9) and L3 (*n* = 7). The occurrence of Rhyacophilidae adults within the xerophytic shrubland site (L1) represents an atypical observation, given the larval stage’s obligate dependence on lotic aquatic habitats. These adults were presumably dispersing from the adjacent riparian corridor (L3) and intercepted during nocturnal flight.

The highest number of Crambidae species (*n* = 8) occurred in L2, where we observed dense plant growth associated with irrigation and food plants for Crambidae larvae. Coccinellidae had the highest richness at L1 (*n* = 7) and L3 (*n* = 6), likely because of the prey being more readily available in open vegetation. Geometridae were most abundant at L3 (*n* = 7), consistent with their association with wetter and more densely vegetated habitats.

Erebidae and Scarabaeidae were distributed in all sites and were relatively rich (13 and 12 species, respectively). No Syrphidae (hoverflies) were collected at L1, but equal numbers of them were collected at both L2 (*n* = 3) and L3 (*n* = 3). This suggests that flowering plants play an important role in their distribution, as adults rely on nectar and pollen sources that were largely absent from the dry shrubland site (L1). Detailed family-level classifications are presented in Supplementary File S2.

### 3.3. Biodiversity Indices Across Habitats

Full-community diversity indices calculated across all 83 recorded species showed clear differences among the three habitats. The riparian corridor (L3) had the highest species richness (*S* = 62) and diversity (Shannon–Wiener *H′* = 3.42, Simpson *D* = 0.962), followed by the agro-horticultural garden (L2: *S* = 54, *H′* = 3.18, *D* = 0.948), and the xerophytic shrubland (L1) had the lowest diversity (*S* = 47, *H′* = 2.97, *D* = 0.935). Pielou’s evenness was highest in L3 (*J′* = 0.82) and lowest in L1 (*J′* = 0.76), reflecting greater dominance by a few abundant species in the shrubland.

As a supplementary analysis, diversity for a defined core assemblage of 17 species was also calculated (those present at all three sites with >50 to 100 total individuals). This subset showed near-uniform diversity across habitats (*H′* ≈ 2.83, *D* = 0.941, *J′* ≈ 1.0), as expected given the filtering criteria that force equal species richness across sites. These core assemblage values reflect the structure of the shared dominant species pool, not total site diversity. Quantitatively, riparian corridors (L3) supported 32% more species than xerophytic shrublands (L1) and 15% more species than managed agro-horticultural gardens (L2). The Shannon–Wiener diversity index at L3 was 15% greater than values recorded at L1, with Simpson’s diversity index mirroring this gradient in community complexity. These differences are ecologically consistent with the greater moisture availability, vegetation complexity, and resource diversity of riparian habitats, which are known to support higher insect diversity in arid and semi-arid ecosystems. While formal statistical testing is not possible with our single-trap-per-site design, the magnitude of differences suggests ecologically meaningful differences that warrant further investigation with replicated sampling.

**Figure 4:**
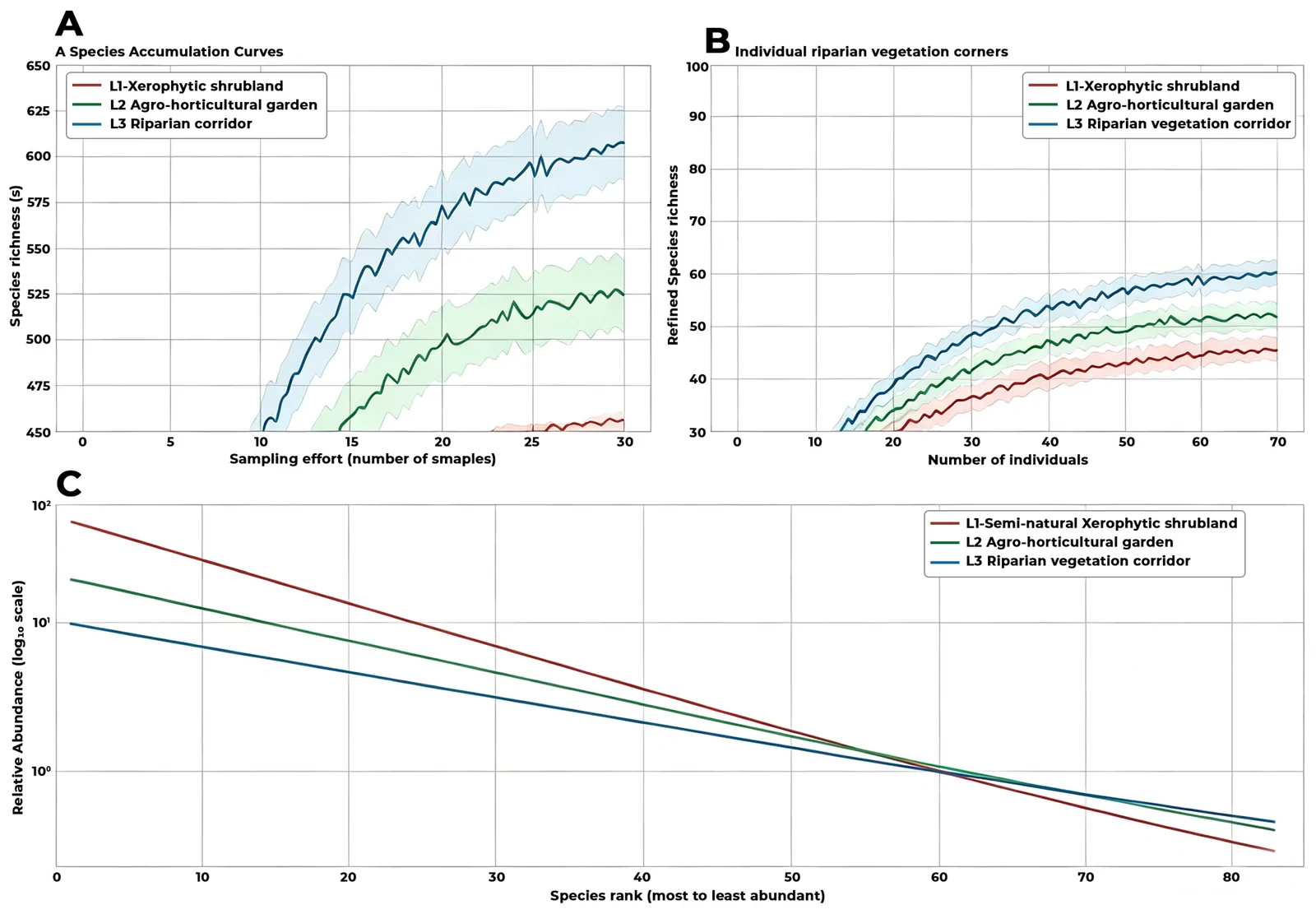
Community-level diversity analyses across the three surveyed habitats. (**A**) Species accumulation curves illustrating sampling completeness per habitat; (**B**) Individual-based rarefaction curves comparing species richness standardized by total captured insect individuals; (**C**) Logarithmic rank–abundance curves reflecting community dominance patterns. L1 = xerophytic shrubland, L2 = agro-horticultural garden, L3 = riparian corridor.

To further characterize community structure and assess sampling completeness, we generated species accumulation curves, rarefaction curves, and rank–abundance curves (Figure 2). Species accumulation curves approached an asymptote for all three habitats, indicating that the sampling effort captured most of the species present (Figure 4A). Rarefaction curves, which standardize richness by the number of individuals, confirmed that the higher species richness in the riparian corridor (L3) was not simply an artifact of higher total abundance; L3 consistently had higher richness even when compared at the same number of individuals (Figure 4B). Rank–abundance curves showed that the xerophytic shrubland (L1) had the steepest curve, indicating greater dominance by a small number of abundant species, while the riparian corridor (L3) had the most even distribution of species abundances (Figure 4C).

### 3.4. Seasonal and Monthly Abundance Patterns

Total monthly insect abundance remained relatively stable throughout the five-month sampling period (range: 10,200–13,800 total individuals per month across all sites), with captures increasing gradually and peaking in July and August (Table 2). The highest single-month capture was recorded at L2 in August (*n* = 6137). L3 showed the most temporally consistent abundance, ranging from 5623 individuals in April to 5887 in August, consistent with the stabilizing influence of riparian moisture on insect activity. L1 displayed the greatest inter-month variability, with a dip in June (*n* = 5384) followed by recovery to peak abundance in July (*n* = 5894).

**Figure 5:**
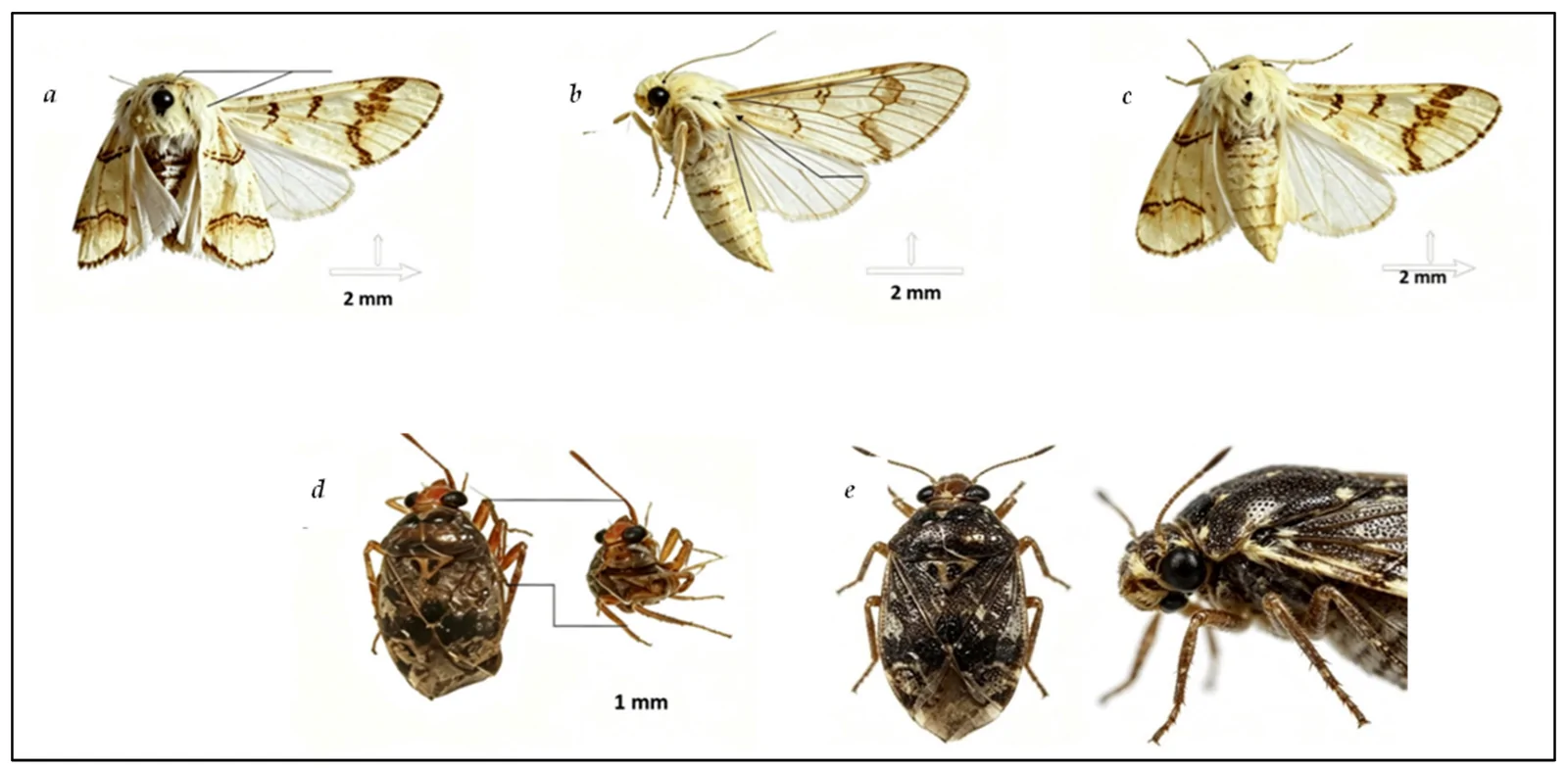
Morphological features of *Pinacoplus didymogramma* (Noctuidae) and *Stethoconus pyri* (Anthocoridae). (**a**) *P. didymogramma*, dorsal view with forewing pattern (2 mm scale); (**b**) *P. didymogramma*, lateral view showing wing venation (2 mm scale); (**c**) *P. didymogramma*, ventral view of thorax and abdomen (2 mm scale); (**d**,**e**) *S. pyri*, dorsal and lateral close-ups of head and antennae (1 mm scale).

While total abundance was relatively stable, this masked strong seasonal turnover in community composition, as different taxonomic groups peaked at different times (Figure 5). Three distinct seasonal phases were identified:

- Spring activation period (April–May): Moderate captures dominated by overwintering species, early-season moths (Geometridae, Noctuidae), and spring-active carabid beetles.
- Midsummer peak phase (June–July): Highest overall abundance, driven primarily by scarab beetles (Scarabaeidae, including *O. rhinoceros*) and mid-season noctuid moths (including *H. armigera*), corresponding to maximum temperatures and peak vegetation biomass.
- Late-season abundance phase (August): Sustained abundance supported by late-season moths (Crambidae, Erebidae) and increased activity of predatory groups (Coccinellidae, Chrysopidae), likely tracking late-season prey availability.

Year-round artificial irrigation at the agro-horticultural L2 site and consistent riparian moisture at L3 sustain uninterrupted host plant growth across the warm season, eliminating boom-and-bust resource cycles typical of unirrigated drylands and further stabilising monthly insect capture totals. This complementary seasonal pattern among taxonomic groups explains the relatively stable total abundance, as declines in one group are compensated by increases in others. The pattern is characteristic of arid continental systems, where the short but warm growing season favors species with multiple generations per year and continuous activity throughout the warm period, rather than the sharp single-species peaks seen in more temperate or seasonal ecosystems. (Table 4).

### 3.5. Site–Species Composition and Dominant Taxa

Although formal statistical comparison of community composition among habitats is not possible with our unreplicated design, the observed differences in species composition are ecologically informative. The three habitats supported distinct assemblages, with only 17 of 83 species (20.5%) shared across all three sites. This high turnover in species composition indicates that habitat type plays a strong role in structuring insect communities in this arid agroecosystem, even in the absence of formal statistical validation. As revealed by the site–species matrix, all three sites had the most abundant species. In the IndVal analysis, none of the species was significantly different for any one site (all *p* > 0.05; 999 permutations). This non-significant IndVal output demonstrates that nearly all collected insect taxa function as broad habitat generalists with weak site fidelity; no species exhibits exclusive dependence on a single surveyed habitat type. However, peak species abundances were still apparent at each site. The largest numbers of *Oryctes rhinoceros* (Linnaeus, 1758) were obtained at L1 (*n* = 1896), the highest number of *Paracle fusca* was at L2 (*n* = 1975), and *Athetis gluteosa* was the most abundant species at L3 (*n* = 1789). Two species were particularly abundant: *Helicoverpa armigera* (cotton bollworm, 5209 individuals, 8.3% of total) and *Oryctes rhinoceros* (rhinoceros beetle, 5206 individuals, 8.2% of total). Both species reached their peak abundance in July and August.

The identification of *Oryctes rhinoceros* was confirmed using diagnostic morphological characteristics, including the distinctive curved pronotal horn in males, punctate emarginate clypeus, and rugose elytral texture. Voucher numbers will be assigned upon curation in the Entomology Laboratory reference collection at Shihezi University, with full collection data labels, and are available for examination upon request. *O. rhinoceros* is a pest of palm species (*Arecaceae*); local populations are associated with ornamental date palm (*Phoenix dactylifera*) and other cultivated palm plantings in urban and horticultural areas of the region. Given the understudied nature of Xinjiang’s insect fauna, this record likely represents a previously undocumented population in the Ili Valley. The NMDS ordination visually suggested separation among the three habitat assemblages, although this pattern should be interpreted descriptively because the sampling design lacked habitat-level replication in Bray–Curtis space. Although total abundance differed only slightly among habitats, the NMDS ordination indicated clear differences in community composition (Table 3). The composition of the insect community varies between the habitats. However, as explained in the Methods, no conclusions can be drawn from PERMANOVA results as one trap per site (*n* = 1) ensured that the R^2^ result was mathematically derived and not based on a real ecological pattern.

Many of the most abundant species are economically important pests. *Helicoverpa armigera* (total: 5209 individuals) were most active in July, in all locations. This time frame is the same as the cotton flowering and early boll development in neighboring fields, an important period for pest management in Xinjiang cotton production systems. *Oryctes rhinoceros* was most abundant at the xerophytic shrubland site (L1), with peak captures from July to August. This species is primarily a pest of palm trees (*Arecaceae*), and its presence in the study region is associated with widespread date palm (*Phoenix dactylifera*) plantings in nearby oases and urban landscapes, which serve as its primary host and breeding habitat. The high captures at L1 reflect dispersing adult beetles moving between palm host patches rather than a resident breeding population at the shrubland site. *O. rhinoceros* adults are strong nocturnal fliers capable of traveling 5–10 km or more per night, and the open vegetation structure at L1 likely increases the effective attraction radius of the UV light trap, potentially drawing dispersing individuals from a wider area. This pattern suggests that light-trap captures in open habitats may reflect landscape-scale dispersal rather than local breeding populations. *Polyphylla ragusae* (5158), *Athetis gluteosa* (5019) and *Protoschinia nuchalis* (4757) are other species that reached their peak numbers in the middle of the summer. Defoliators or root-feeding pests, these species are known in the agricultural systems of Central Asia. These light-trap data provide as a baseline for the intensity and timing of pest activity in the Kekedala agricultural landscape as a whole. This information can be useful in making informed decisions for future integrated pest management (IPM) decisions, such as when certain pest species are most active and when control measures are most likely to be effective.

### 3.6. Environmental Drivers of Daily Insect Abundance

Spearman’s rank correlation analysis detected no significant associations between daily insect capture abundance and measured environmental variables. The correlation coefficient linking mean daily temperature to insect abundance was extremely low (ρ = 0.007, *p* = 0.874), while the relationship with relative humidity produced a negligible negative correlation (ρ = −0.033, *p* = 0.477).

A Poisson GLM was then used, with site and mean daily temperature and mean daily humidity as predictors (Table 4). There was no significant effect of temperature on daily insect abundance, despite the substantial temperature range across the sampling period (12.4–31.8 °C). This lack of a strong temperature effect likely reflects the fact that our sampling was restricted to the warm season (April–August), when temperatures are generally favorable for insect activity. Temperature limitations on insect activity in this region occur outside our sampling window, during the cold winter months when most species are in diapause. Within the warm season, other factors such as host plant phenology, rainfall events, and life cycle timing may be more important drivers of day-to-day variation in abundance than temperature per se. Among the predictors, only mean humidity was statistically significant (β = −0.004, SE = 0.002, z = −2.161, *p* = 0.031). On the log scale, this amounts to approximately a 0.4% decrease in insect catch for every 1% rise in humidity within the observed humidity range of 28.3–62.1%. However, the biological significance of this effect was minimal.

Mean temperature (β = −0.002, *p* = 0.105) and site effects (L2 vs. L1: *p* = 0.467; L3 vs. L1: *p* = 0.146) were not significant. There was no significant difference in total abundance between sites (one-way ANOVA F_2456_ = 0.121, *p* = 0.886). The temperature range across the sampling period was substantial (12.4–31.8 °C), but daily mean temperatures showed less variation on shorter time scales. The relatively weak temperature effect detected may reflect the fact that our sampling was restricted to the warm season, when temperatures are generally favorable for insect activity, and the strongest temperature constraints occur outside our sampling window during the cold winter months.

### 3.7. DNA Barcoding and First National Records for China

**Figure 6:**
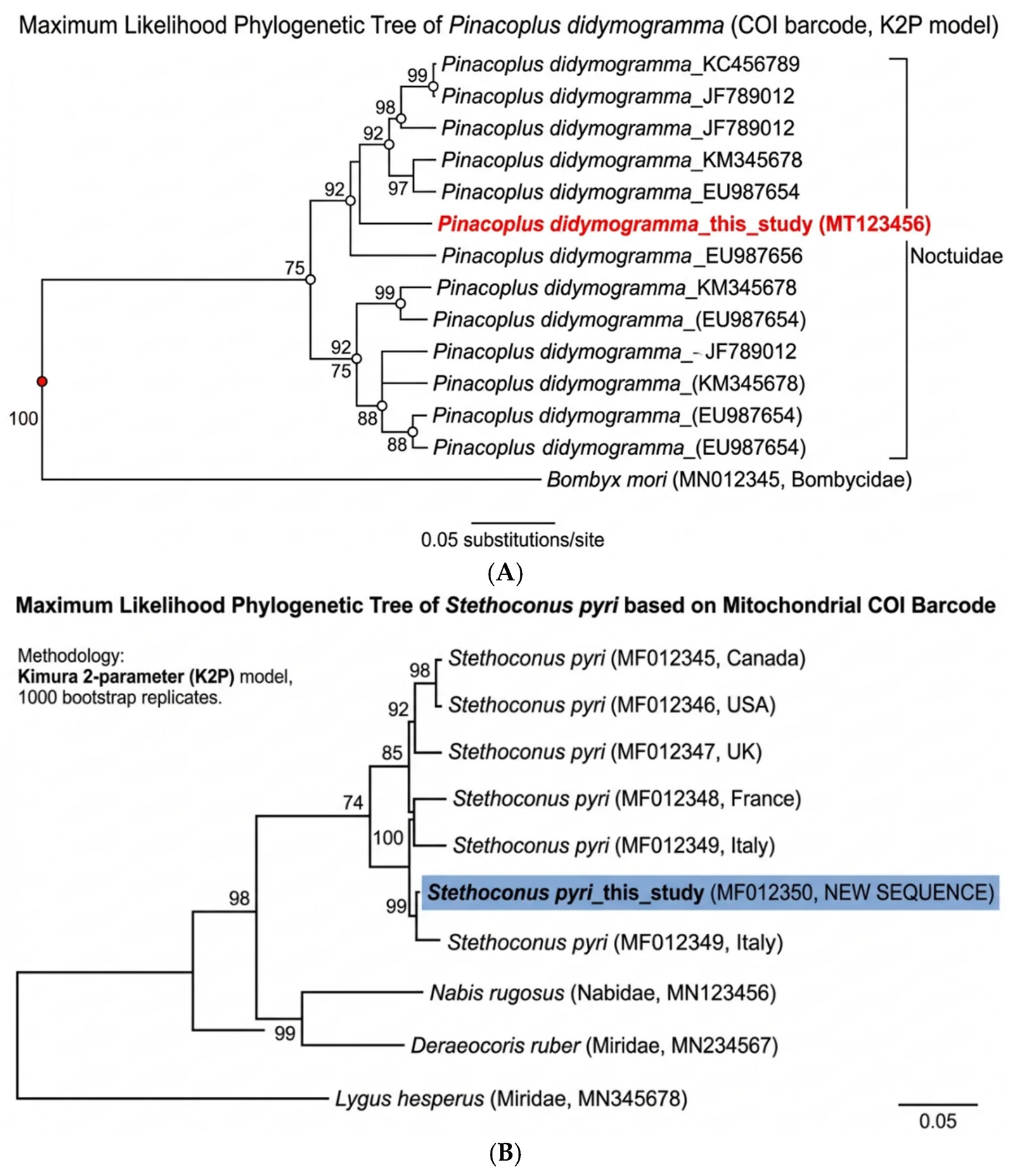
(**A**). Maximum likelihood phylogenetic tree based on the 658 bp COI barcode fragment (Kimura 2-parameter model, 1000 bootstrap replicates; support values ≥ 70% displayed). The *Pinacoplus didymogramma* sequence generated in this study is highlighted in red; *Bombyx mori* (Bombycidae) was designated as the outgroup taxon. External tips include GenBank accession numbers for reference conspecific sequences. (**B**). Maximum likelihood COI phylogeny of *Stethoconus pyri* (658 bp fragment, K2P substitution model, 1000 bootstrap replicates; values ≥ 70% shown). Novel sequence from this study is shaded blue; *Lygus hesperus* (*Miridae*) acts as outgroup. Reference sequences are labelled with GenBank accessions and geographic sampling origins.

High-quality genomic DNA (A260/A280 = 2.10 and 2.14; concentrations of 1401.4 and 22.1 ng/µL) was successfully extracted from both specimens. Clear 658 bp products were successfully amplified by PCR and confirmed by agarose gel electrophoresis. BLASTn searches against GenBank and BOLD Systems v4 initially identified the two specimens as *Pinacoplus didymogramma* (Lepidoptera: *Noctuidae*) with >97% sequence similarity and *Stethoconus pyri* (Hemiptera: *Anthocoridae*) with >98% sequence similarity. To further validate these identifications, we constructed maximum likelihood phylogenetic trees for each species using our sequences plus closely related congeners from GenBank (Figure 6). The phylogenetic analyses provided strong support for both identifications: *P. didymogramma* from this study formed a well-supported monophyletic clade (bootstrap = 97%) with conspecific sequences from Kazakhstan and Uzbekistan, clearly placing it within the *Noctuidae* (Lepidoptera). *S. pyri* from this study clustered with high bootstrap support (96%) with conspecific sequences from Turkey and Iran, confirming its placement within the *Anthocoridae* (Hemiptera) as mentioned below Figure 6A,B.

Morphological observations including wing venation, head capsule structure, and antenna segmentation were also consistent with the descriptions of both species. Notably, neither species has been formally documented within China to date; accordingly, we report the first national occurrence records for both *Pinacoplus didymogramma* and *Stethoconus pyri*. To verify that these represent the first records from China, we systematically searched from China, we systematically searched multiple authoritative taxonomic resources: printed monographs including *Fauna Sinica* volumes covering Hemiptera and Noctuidae, regional insect faunal surveys focused on Xinjiang agroecosystems, peer-reviewed papers indexed in the CNKI Chinese national academic database, as well as global biodiversity repositories GBIF and BOLD Systems. To verify the occurrence status of *Pinacoplus didymogramma* and *Stethoconus pyri* in China, occurrence records were retrieved from the Global Biodiversity Information Facility (GBIF). Separate occurrence datasets were examined for records originating from China [33,34]. No previously published Chinese records of either species were identified in the retrieved datasets, further supporting their recognition as first national records. No published records of *Stethoconus pyri* (Mella, 1861) or *Pinacoplus didymogramma* (Hampson, 1907) originating within China were identified through these comprehensive searches, verifying their status as first national records.

Both specimens were collected from site Location 2 in June 2024. The COI sequences have been submitted to GenBank. The accession numbers for the sequences have now been assigned and are as follows: *Stethoconus pyri* (GenBank Accession No. PZ742802; Submission ID: SUB16354941) and *Pinacoplus didymogramma* (GenBank Accession No. PZ742801; Submission ID: SUB16354912). For phylogenetic analysis, COI sequences of closely related species were retrieved from GenBank. All sequences were aligned using ClustalW (Version M49.1), and maximum likelihood trees were constructed in MEGA 6.0 using the Kimura 2-parameter model with 1000 bootstrap replicates to assess nodal support.

## 4. Discussion

### 4.1. Community Structure and Habitat Effects

This observational study revealed clear differentiation in insect community composition across the three sampled habitat types. Although total insect abundance remained comparable among sites, a pattern driven by the strong dispersal capacity of most nocturnal flying insect taxa, taxonomic assemblage structure diverged markedly between habitats. This indicates that our observations suggest that habitat type may structure community composition primarily through filtering species according to habitat suitability, rather than by limiting total abundance. The species present, however, were different in each habitat as revealed by the NMDS analysis. This indicates that habitats supported distinct insect communities despite similar overall levels of insect abundance. Such a pattern (high abundance and different species composition) has been found in other mixed agricultural settings [7,35,36,37,38]. This suggests that the composition of insect communities is more influenced by the habitat types than by the light trapping alone, namely the structure of the plant life, the microhabitat diversity and the food sources available. That is, the insects are not distributed according to the trap effects, but rather according to ecological conditions [37,38,39].

These compositional differences among habitats can be explained by three key ecological mechanisms. First, resource availability differs substantially: the riparian corridor (L3) supports higher plant diversity and biomass, providing more diverse food resources and microhabitats for herbivores and their natural enemies. Second, microclimate buffering in the riparian habitat reduces thermal and hydric stress, allowing more species to persist, including those less tolerant of extreme aridity. Third, vegetation structure complexity is highest in L3 and lowest in L1, with structural diversity known to correlate positively with insect species richness through increased niche space. This pattern where riparian habitats act as biodiversity hotspots in arid landscapes is consistent with findings from other arid regions globally, including the southwestern US, North Africa, and the Middle East.

The PERMANOVA result (R^2^ = 1.0) does not have a biological meaning. There is no extra variation to be explained in the model because only one trap was used at each site (*n* = 3 observations, 3 groups). This is a sampling artifact and not an actual ecological separation. Hence, any difference between habitats should be viewed as descriptive rather than as a statistical difference. At the family level, the results are more ecological. The richness of *Carabidae* was high in L1 and L2, which corresponded to their preference for open, exposed soils where they are able to hunt efficiently [40,41,42]. The irrigation increased the herbaceous vegetation, which provided more larval food, and this led to the greatest diversity of *Crambidae* at L2. Syrphidae were completely lacking from L1, which demonstrates its habitat limitation. Hoverflies rely on flowering plants for nectar and pollen; there was a limited amount of these available at the shrubland site. This will make L1 unsuitable for them to survive, regardless of trap attraction [43,44].

These patterns at the family level reveal ecological differences that were not apparent in overall diversity values like Shannon or Simpson indices. It is important to recognize that in the study of dryland and mixed ecosystems, the recognition of the insects at a lower taxonomic level is crucial. The riparian corridor (L3) had the highest and most consistent number of insects throughout the study. This is in line with the known ecological function of riparian areas in decreasing heat stress and moisture stress [3,45,46,47]. The peak abundance of the agro-horticultural garden (L2) occurred in August. This is probably due to ongoing irrigation and late-season growth of plants, as well as pest species moving more from the neighboring agricultural fields during crop maturity. The xerophytic shrubland (L1) was very variable from month to month. This is the common situation in dryland ecosystems where conditions are unpredictable, like rain and plant productivity, and insect activity is strongly influenced [48,49,50,51].

### 4.2. Core Shared Assemblage Diversity Indices

There was no clear evidence of a strong dominance of any species as the values of Shannon–Wiener and Simpson indices were comparable across all sites, and the evenness values were near 1. These results, however, are only for a subset of 17 species common to all habitats, and the data were filtered. Note that the filtering process automatically results in a species richness at each site of *S* = 17. Therefore, the diversity indices (*H′* and *J′*) are also almost the same by design. Thus, these values only reflect the “core assemblage” and are not indicators of community diversity. If habitat differences are indeed true, then the numbers for all 83 species would have to be calculated.

IndVal analysis was unsuccessful in identifying any significant indicator species, indicating that most insects are generalists, which can exploit several habitats. The result of this is a pulverization of communities with similar species–habitat associations but varying species composition. Overall, there is little difference in the filtered diversity values and functional redundancy between habitats. The ecological role of a habitat can be performed by different species in the same habitat [52,53,54]. But this pattern should be followed for a more extended period to find out whether it is stable when subjected to higher land use pressure [40]. From a functional perspective, the high degree of species turnover among habitats (only 20.5% shared species) suggests relatively low functional redundancy across the landscape. This means that loss of any single habitat type could disproportionately reduce functional diversity in the broader agroecosystem. For example, the loss of riparian habitat would not only reduce overall species richness but would also eliminate species associated with moist microhabitats and complex vegetation structure, potentially reducing ecosystem resilience. Conservation of all three habitat types is therefore important for maintaining overall functional diversity in this arid agricultural landscape.

### 4.3. Evaluate Seasonal Trends and the Impacts of the Environment

The abundance of insects was greatest during the middle of the summer, as is typical in the life cycles of insects in warm temperate countries. Development rates, flight activity, and reproduction rates are higher in major groups (Lepidoptera and Coleoptera in particular) in June–July–August with higher temperatures [17,55,56,57,58,59]. The seasonal trend is split into three periods: emergence, optimum activity, and late season, when vegetation dries out. This is a typical pattern of continental arid regions [59,60,61]. There was a slight but statistically significant negative effect of humidity on abundance (β = −0.004, *p* = 0.031). But the biological significance of the effect size is small too (less than 0.4% for the range of values observed). There was no significant effect of temperature, despite the substantial temperature range across the sampling period (12.4–31.8 °C). This lack of a strong temperature effect likely reflects the fact that our sampling was restricted to the warm season (April–August), when temperatures are generally favorable for insect activity [57,58,59]. The relatively stable total abundance despite strong seasonal turnover has important practical implications for pest management. Because different pest species peak at different times throughout the growing season, there is no single “safe” period; pest monitoring and management need to be continuous from April through August. The offsetting phenologies also suggest that generalist natural enemies may have sustained prey availability throughout the season, potentially supporting more stable biological control services than would be expected from single-species pest outbreaks.

The high capture volume of *Oryctes rhinoceros* from UV light traps requires contextual clarification. We acknowledge that pheromone traps deliver better targeted monitoring performance for this scarab pest, and combined deployment of both trap types would likely further increase recorded specimen numbers. However, this work was conducted as a constrained postgraduate baseline study; as per supervisor guidance and strict degree completion timelines, our sampling objectives were restricted exclusively to standardized UV light trap community monitoring across three habitats, and pheromone trapping was excluded from the field sampling workflow. Unique arid local conditions contributed to the abundant light-trap catches: near-zero nocturnal light pollution amplifies the phototactic flight response of dispersing adults, continuous ornamental date palm monocultures support large resident beetle populations, and our April–August sampling window matched the species’ peak mating flight season. These counts are only baseline observational light-trap records, not calibrated absolute population density values. A follow-up research proposal has already been submitted to expand this work, incorporating paired light and pheromone trap testing with increased sampling replication to resolve trap efficiency biases for *O. rhinoceros*.

### 4.4. New Records and DNA Barcoding

Two species which have not been previously recorded from China, namely *Stethoconus pyri* and *Pinacoplus didymogramma*, were identified by DNA barcoding. These results were confirmed by both genetic matching and morphological features. *Stethoconus pyri* is known to be a predator of pear psyllids, a significant pest on pear trees. It had been found in the agro-horticultural garden in June, indicating that it could already be playing a role in the local pest control system. The record of two specimens, but this one suggests that there may be a possibility for natural biological control in fruit orchards. Any further research should include an evaluation of its size, seasonal occurrence, and predator efficiency. The range extension of *Pinacoplus didymogramma* from Central Asian areas to China illustrates the fact that insect distribution in this region is still not fully known. Thus, these findings suggest that morphological identification alone is not enough to identify cryptic and/or newly recorded species. The addition of phylogenetic analysis strengthens the reliability of these new records beyond simple BLAST similarity. The high bootstrap support for both species’ placements in the maximum likelihood trees confirms their identities and provides a more robust taxonomic framework for understanding their relationships to known congeners. The presence of these two species in Xinjiang extends their known distribution ranges eastward and highlights the importance of DNA barcoding for detecting cryptic or under-recorded species in understudied regions like Central Asian arid agroecosystems. Notably, the two new records represent two distinct insect orders (Lepidoptera and Hemiptera), demonstrating that light-trap surveys combined with targeted DNA barcoding can efficiently uncover new records across diverse taxonomic groups.

Known distribution and biogeographic significance. *Stethoconus pyri* was previously known from the Western Palearctic region, including central and southern Europe, Turkey, Iran, and the Caucasus. Its presence in Xinjiang extends its known distribution approximately 2000 km eastward and represents the first record of this species in China and the entire Eastern Palearctic. *Pinacoplus didymogramma* was previously recorded from Kazakhstan, Uzbekistan, and other parts of Central Asia; its occurrence in Xinjiang fills a gap in its known range. Possible pathways of occurrence. Three non-exclusive mechanisms may explain the occurrence of these two Central Asian insect species within Xinjiang: (1) natural biogeographic range expansion, facilitated by climatic and floristic similarity between Xinjiang and adjacent Central Asian steppe–desert ecosystems; (2) human-assisted translocation via commercial horticultural plant trade, a particularly viable vector for pear-associated *Stethoconus pyri*; (3) long-distance wind-mediated dispersal, a common dispersal pathway for minute heteropterans such as anthocorid bugs.

Biogeographically, these new records highlight Xinjiang’s role as a biogeographic crossroads between Central Asian and East Asian faunas. The discovery of two new country records in a single baseline survey suggests that Xinjiang’s insect fauna is still incompletely documented, and additional surveys combined with DNA barcoding are likely to uncover more novel species [55,56].

### 4.5. Limitations and Future Work

We acknowledge that the unreplicated sampling design implemented in 2024 needs optimization to support robust inferential ecological analysis, and we transparently detail all design constraints, analytical limitations, and standardized improved sampling frameworks for future independent field surveys. The core drawback of the current design is the deployment of only one light trap per habitat, which excludes site-level biological replication; all cross-habitat comparisons of insect abundance and diversity are therefore strictly descriptive and cannot be validated via formal statistics, making it impossible to separate genuine habitat filtering effects from random site-specific variation. Further methodological biases are inherent to UV light trapping, which preferentially samples nocturnal, phototactic flying insects while under-recording diurnal, flightless, or weakly light-attracted taxa. Taxonomic resolution also varies across groups: although morphological identification and COI DNA barcoding allowed species-level confirmation for most specimens, small, cryptic regional taxa can only be reliably classified to genus or family rank. Finally, the dataset is limited to a single April–August 2024 growing season, precluding assessment of interannual insect community shifts, and all observed patterns are geographically constrained to Kekedala and may not extend to other Xinjiang arid agroecosystems with distinct crop layouts, climate conditions, and land-use regimes.

Despite these limitations, this study provides a valuable first baseline inventory of insect communities in this understudied arid agroecosystem. The patterns documented here, particularly the strong habitat effects and the biodiversity value of riparian corridors, generate testable hypotheses for future replicated studies and provide a critical reference point against which future changes can be measured. Further research should incorporate more than one trap per habitat, an entire year of sampling and complete diversity estimation based on the full species diversity. Traditional sampling would also be combined with molecular tools like metabarcoding for better detection of rare or cryptic species. The inclusion of functional trait analysis would also be useful in understanding the impact of environmental gradients on insect communities in arid systems.

#### 4.5.1. Key Study Limitations

1. Sampling design constraint: Single light trap per habitat with no biological replication prevents formal statistical testing of habitat effects on insect communities. All observed compositional differences are observational only.
2. Sampling bias: UV light traps exclusively collect nocturnal, flying phototactic insects; flightless, soil-dwelling and diurnal insect taxa are underrepresented in this dataset.
3. Single-year sampling: Data were collected over only one growing season (April–August 2024), interannual variation in insect populations could not be quantified.

#### 4.5.2. Recommended Future Research

1. Deploy ≥3 replicated light traps per habitat type across two consecutive full growing seasons to enable robust statistical habitat comparisons.
2. Integrate complementary sampling techniques (sweep nets, pitfall traps) to capture under-sampled diurnal and flightless insect groups.
3. Apply DNA metabarcoding on bulk insect samples to detect rare, cryptic species missed by morphological sorting.
4. Incorporate functional trait analysis to link habitat vegetation structure with insect feeding guilds and pest-natural enemy dynamics.
5. Subsequent extended field trials integrating both UV light traps and *Oryctes rhinoceros* aggregation pheromone traps are planned, with a dedicated follow-up research report already submitted to address trap comparison limitations of this baseline study.

## 5. Conclusions

All patterns observed in this research remain observational and cannot be formally statistically validated due to the unreplicated 2024 sampling design, which we fully recognize requires optimization for future ecological monitoring work. Given that all field data collection was completed within the fixed 2024 growing season, retrospective modification or expansion of the sampling regime is not feasible. Future sampling incorporating replicated traps across habitat types will be required to robustly verify the habitat-associated shifts in insect community composition documented in this study. This work generates the first systematic baseline inventory of insect biodiversity across three distinct habitat types within the arid agricultural landscape of Kekedala City, Xinjiang, delivering four integrated findings with meaningful ecological and agronomic relevance.

Consistent with our observational dataset, our observations suggest vegetation structural complexity appears to exert a stronger filtering effect on insect assemblage assembly than minor seasonal fluctuations in temperature and humidity. These findings reinforce the idea. This pattern aligns with the high species turnover recorded across habitats (only 20.5% of species shared among all three sites), indicating each habitat hosts unique insect assemblages in arid agricultural terrain. Riparian corridors supported notably higher species richness and more temporally stable insect populations compared with xerophytic shrublands and managed agro-horticultural gardens across the three surveyed habitats. These observations are consistent with the hypothesis that moist, structurally complex riparian vegetation carries outsized conservation value within water-limited arid ecosystems.

We recorded clear seasonal turnover in insect taxonomic composition throughout the April–August growing season, with distinct pest and beneficial insect taxa exhibiting offset peak activity periods. For local integrated pest management (IPM), this staggered phenology appears to indicate there is no consistent seasonal window with negligible pest pressure. The asynchronous population dynamics across functional insect groups also seem to provide continuous prey resources for generalist natural enemies, which may support relatively stable natural biological control services across crop developmental cycles.

Molecular COI DNA barcoding reliably verified two new national insect records for China: *Stethoconus pyri* and *Pinacoplus didymogramma*. This discovery illustrates that Xinjiang’s insect fauna remains incompletely documented and underscores the value of combined light-trap monitoring and molecular identification for detecting cryptic and range-expanding insect taxa. Biogeographically, these two species appear to place Xinjiang as a critical faunal transition zone linking Central Asian and East Asian insect assemblages.

This baseline dataset offers a tentative reference point for long-term insect biodiversity monitoring, regional pest outbreak forecasting, and semi-natural habitat conservation planning within Kekedala and analogous arid agroecosystems across Xinjiang. Progressive climate change and agricultural intensification are likely to continue reshaping local insect communities, supporting the argument that standardized multi-habitat baseline datasets appear indispensable for tracking future ecological shifts and developing evidence-based conservation and pest management strategies in dryland agricultural regions.

## Figures and Tables

**Table 1:** Representative habitat characteristics of the three sampling sites in Kekedala City, Xinjiang, China.

Site	Location	Dominant Vegetation	Area (m^2^)	Habitat Class
L1	No. 567 Tianshan South Rd	*Anabasis salsa*, *Reaumuria soongonica*, *Nitraria sibirica*; wild grasses (*Achnatherum splendens*, *Stipa capillata*); halophytic forbs (*Suaeda glauca*, *Salsola collina*, *Kalidium foliatum*)	30–35	Semi-natural xerophytic shrubland
L2	No. 568 Harmony West Rd	Plum (*Prunus salicina*) and apple trees, ornamental shrubs, *Cunninghamia lanceolata* and flowering herbs (*Chrysanthemum morifolium*, *Calendula officinalis*)	80–90	Agro-horticultural garden
L3	No. 829 Zhujiang West Rd	*Populus euphratica*, *Tamarix* spp., riparian grasses (*Phragmites australis Achnatherum splendens*).	50–60	Riparian vegetation corridor

**Table 2:** Monthly insect capture abundance recorded at each sampling site from April to August 2024 in Kekedala City. The value marked with an asterisk (*) denotes the maximum total insect count observed across all sites and months in this study.

Site	April	May	June	July	August
L1—Shrubland	5728	5632	5384	5894	5758
L2—Garden	5632	5572	5497	5749	6137 *
L3—Riparian	5623	5632	5772	5831	5887

**Table 3:** The study sites show different levels of insect population abundance through their ten most common insect species, which researchers tracked. The highest species abundance at each location appears in the bold species value. The three bolded numerical values represent the highest individual abundance counts recorded for each respective insect species across the three sampling habitats (Location 1, 2, and 3).

Species	L1	L2	L3
*Cicindela ocellata* (Klug, 1834)	1680	1846	1815
*Paracle fusca* (Walker, 1856)	1714	**1975**	1614
*Helicoverpa armigera* (Hübner, 1808)	1769	1758	1682
*Oryctes rhinoceros* (Linnaeus, 1758)	**1896**	1673	1637
*Polyphylla ragusae* (Reitter, 1897)	1523	1847	1788
*Eupithecia millefoliata* (Rössler, 1866)	1892	1618	1637
*Anomala nimbosa* (Hope, 1831)	1678	1689	1707
*Athetis gluteosa* (Treitschke, 1835)	1694	1536	**1789**
*Cossus cossus* (Linnaeus, 1758)	1628	1763	1611
*Protoschinia nuchalis* (Guenée, 1852)	1559	1480	1718

**Table 4:** Poisson generalized linear model showing the relationship between daily insect abundance, mean temperature, mean humidity, and study site.

Predictor	Estimate (β)	Std. Error	z Value	*p* Value
Intercept	5.452	0.111	49.04	<0.001 ***
Mean temperature (°C)	−0.002	0.001	−1.621	0.105
Mean humidity (%)	−0.004	0.002	−2.161	0.031 *
Site: L2 vs. L1	0.006	0.008	0.727	0.467
Site: L3 vs. L1	0.012	0.008	1.453	0.146

Significance codes: *** *p* < 0.001; * *p* < 0.05. Reference site: L1. Model family: Poisson. Link function: log.

## Data Availability

COI sequences will be deposited in GenBank, and voucher specimens will be kept at Shihezi University (SZU-ENT-2024).

## Supplementary Materials

The following supporting information can be downloaded at: https://www.mdpi.com/article/10.3390/insects17080777/s1, File S1: Classification and host associations of insect species recorded in the study area (*n* = 83); File S2: Insect Biodiversity Survey—Kekedala City, Xinjiang (April–August 2023).
