# Supplementary material for: Habitat-Driven Insect Community Structure in an Arid Agroecosystem of Xinjiang, China: Integrated Light-Trap Monitoring and DNA Barcoding Evidence
Source: Insects. 2026 Jul 28;17(8):777. doi: 10.3390/insects17080777 (PMC13513039; doi:10.3390/insects17080777)
Supplement: Supplementary file 1 [file insects-17-00777-s001.zip › Supplementary File S1.pdf]

Supplementary Table S1. Classification and host associations of insect species recorded in the study area (n = 83).

Sr.	Species (Authority, Year)	Order	Family	Classification	Host Plants / Damage / Role
			**HIGH-DAMAGE PESTS (17 species)**		
1	*Agrius convolvuli* (Linnaeus, 1758)	Lepidoptera	*Sphingidae*	High-damage pest	Sweet potato (Ipomoea batatas), morning glory (Convolvulaceae): larvae strip foliage rapidly; serious defoliator in tropical and subtropical Asia and Africa.
2	*Plodia interpunctella* (Hübner, 1813)	Lepidoptera	*Pyralidae*	High-damage pest	Stored grain, dried fruit, nuts, cereals, chocolate: larvae contaminate stored products worldwide; Indian meal moth; major stored-product pest in South Asia including Pakistan.
3	*Spodoptera arvensis* (Boisduval, 1833)	Lepidoptera	*Noctuidae*	High-damage pest	Wheat, maize, sorghum, cotton: mass defoliation during outbreak conditions; recorded as a crop threat in Central Asia and the Middle East.
4	*Heliothis viriplaca* (Hufnagel, 1766)	Lepidoptera	*Noctuidae*	High-damage pest	Clover, alfalfa, sunflower, soybean, pea, tobacco: pod and seed borer; recorded pest of legume crops across the Palearctic region.
5	*Opogona sacchari* (Bojer, 1856)	Lepidoptera	*Tineidae*	High-damage pest	Sugarcane, banana, sweet potato, dracaena: larvae tunnel through stems and roots; intercepted quarantine pest in many countries.
6	*Scirpophaga praelata* (Scopoli, 1763)	Lepidoptera	*Crambidae*	High-damage pest	Rice, sugarcane: stem boring causes dead hearts in vegetative stage and white ears at heading; serious pest across South and Southeast Asia.
7	*Agriotes lineatus* (Linnaeus, 1767)	Coleoptera	*Elateridae*	High-damage pest	Potato, wheat, maize, carrot, onion: wireworm larvae feed on roots and underground stems for 3 to 5 years; causes major yield losses in cereal and vegetable crops.
8	*Parahypopta caestrum* (Hübner, 1808)	Lepidoptera	*Cossidae*	High-damage pest	Asparagus, grasses, herbaceous roots: root-boring larvae cause plant wilting and death; recorded in Mediterranean and Central Asian steppe regions.
9	*Euxoa servitus* (Eversmann, 1842)	Lepidoptera	*Noctuidae*	High-damage pest	Wheat, beet, potato, vegetables: cutworm severs seedlings at soil level; a common cutworm across arid and semi-arid agricultural zones.
10	*Leucania comma* (Linnaeus, 1761)	Lepidoptera	*Noctuidae*	High-damage pest	Wheat, rye, barley, pasture grasses: larvae feed on leaves and stems; outbreak populations cause serious defoliation in cereal-growing regions.
11	*Acrobasis consociella* (Hübner, 1813)	Lepidoptera	*Pyralidae*	High-damage pest	Oak (Quercus spp.), apple, pear: larvae spin and skeletonize foliage; recorded pest of fruit orchards and oak woodland in Europe and Western Asia.
12	*Evergestis frumentalis* (Linnaeus, 1761)	Lepidoptera	*Crambidae*	High-damage pest	Cabbage, turnip, mustard, rapeseed: larvae web and consume growing heads; a significant pest of Brassica crops in Europe, Central Asia, and northern Pakistan.
13	*Pseudocleonus cinereus* (Schrank, 1781)	Coleoptera	*Curculionidae*	High-damage pest	Thistle (Cirsium), artemisia, steppe rangeland plants: adults and larvae destroy roots and stems; serious range pest across arid Central Asian steppe.
14	*Oryctes rhinoceros* (Linnaeus, 1758)	Coleoptera	*Scarabaeidae*	High-damage pest	Coconut (Cocos nucifera), oil palm (Elaeis guineensis), date palm (Phoenix dactylifera): adults bore into growing tips killing meristems; major pest of palms in Asia and the Pacific.
15	*Maladera saitoi* (Niijima & Kinoshita, 1927)	Coleoptera	*Scarabaeidae*	High-damage pest	Turf, vegetables, ornamentals, cereals: white grub larvae sever roots underground; adults defoliate at night; recorded in East and Central Asian agricultural areas.
16	*Myelois fuscicostella* (Ragonot, 1887)	Lepidoptera	*Pyralidae*	High-damage pest	Carob (Ceratonia siliqua), pomegranate (Punica granatum), fig, almond: larvae bore inside fruit causing complete internal destruction; serious pest of orchard fruits in the Mediterranean and Middle East.
17	*Polyphylla ragusae* (Costa, 1839)	Coleoptera	*Scarabaeidae*	High-damage pest	Grapevine (Vitis vinifera), fruit trees, nursery stock: grubs feed on roots for 3 to 4 years; adults defoliate; recorded pest of vineyards in South-East Europe and the Balkans.
			MODERATE PESTS (31 species)		
18	*Apamea unanimis* (Hübner, 1813)	Lepidoptera	*Noctuidae*	Moderate pest	Reed (Phragmites australis), sedge (Carex spp.), coarse grasses near grain crops: larval feeding in stems; localized pest of wetland-margin grassland.
19	*Apochemia cinerarius* (Erschoff, 1874)	Lepidoptera	*Geometridae*	Moderate pest	Fruit trees, oak (Quercus spp.), poplar: sporadic defoliation; outbreak potential low; early-spring flier in semi-arid zones of the Palearctic.
20	*Argyresthia notoleuca* Meyrick, 1922	Lepidoptera	*Argyresthiidae*	Moderate pest	Juniper (Juniperus spp.), cypress (Cupressus spp.): needle mining by larvae; damages ornamental conifers and windbreaks in semi-arid regions.
21	*Hyles gallii* (Rottemburg, 1775)	Lepidoptera	*Sphingidae*	Moderate pest	Bedstraw (Galium spp.), willowherb (Epilobium spp.), grapevine foliage: rarely causes economic loss; larvae strip foliage from wild host plants in late summer.
22	*Chlorochlamys chloroleucaria* (Guenée, 1858)	Lepidoptera	*Geometridae*	Moderate pest	Herbaceous plants, clover (Trifolium spp.), wildflowers: minor geometrid feeder on low-growing vegetation in meadow and steppe habitats.

23	*Ascotis selenaria* (Denis & Schiffermüller, 1775)	Lepidoptera	*Geometridae*	Moderate pest	Fruit trees, mulberry (Morus spp.), citrus: localized defoliation; a polyphagous geometrid moth recorded as a minor orchard pest in the Middle East and Central Asia.
24	*Athetis gluteosa* (Treitschke, 1835)	Lepidoptera	*Noctuidae*	Moderate pest	Grasses, meadow plants: minor noctuid damage; larvae feed on grass roots and low vegetation; no major crop damage documented.
25	*Deilephila elpenor* (Linnaeus, 1758)	Lepidoptera	*Sphingidae*	Moderate pest	Grapevine (Vitis vinifera), fuchsia, willowherb (Epilobium spp.): large larvae defoliate host plants locally; rarely causes economic loss in vineyards.
26	*Chrysoteuchia culmella* (Linnaeus, 1758)	Lepidoptera	*Crambidae*	Moderate pest	Pasture grasses, turf: larvae feed on underground grass stems at the base; common pest of lowland pastures and golf turf in the Palearctic.
27	*Cucullia chamomillae* (Denis & Schiffermüller, 1775)	Lepidoptera	*Noctuidae*	Moderate pest	Chamomile (Matricaria spp.), artemisia: damages medicinal herb crops; recorded as a pest of chamomile cultivation in Central and Eastern Europe.
28	*Cucullia syrtana* Staudinger, 1895	Lepidoptera	*Noctuidae*	Moderate pest	Artemisia spp., steppe composites: arid-zone medicinal plant feeder; larvae consume foliage of artemisia in semi-arid and desert-margin habitats.
29	*Cynaeda dentalis* (Denis & Schiffermüller, 1775)	Lepidoptera	*Crambidae*	Moderate pest	Eryngium spp., thistles (Carduus, Cirsium): narrow host range crambid; larvae spin webs on flower heads; no significant crop pest status.
30	*Eublemma purpurina* (Denis & Schiffermüller, 1775)	Lepidoptera	*Erebidae*	Moderate pest	Helichrysum spp. (everlasting flowers), Asteraceae: larvae feed on flower heads; affects dried flower crops and medicinal plant cultivation in Mediterranean zones.
31	*Emmelia trabealis* (Scopoli, 1763)	Lepidoptera	*Noctuidae*	Moderate pest	Bindweed (Convolvulus arvensis), field convolvulus: field margin feeder; larvae confined to Convolvulaceae; a secondary nuisance in arable fields.
32	*Eudonia sudetica* (Zeller, 1839)	Lepidoptera	*Crambidae*	Moderate pest	Mosses, alpine grasses: larvae feed on moss protonemata in montane habitats; minimal crop significance; no agroeconomic damage recorded.
33	*Dyotopasta yumaella* (Kearfott, 1903)	Lepidoptera	*Lecithoceridae*	Moderate pest	Desert shrubs, sagebrush (Artemisia tridentata-type): arid rangeland moth; larvae feed on dry bark and plant debris; no direct crop host documented.
34	*Eupithecia millefoliata* Rössler, 1866	Lepidoptera	*Geometridae*	Moderate pest	Yarrow (Achillea millefolium), medicinal Asteraceae: host-specific geometrid; damages medicinal herb plantations of yarrow in Central and Eastern Europe.
35	*Drasteria mirifica* (Butler, 1878)	Lepidoptera	*Erebidae*	Moderate pest	Steppe grasses, sagebrush (Artemisia spp.): sporadic rangeland moth; larval ecology not fully described; no direct crop pest status in literature.
36	*Paracle fusca* (Walker, 1855)	Lepidoptera	*Erebidae*	Moderate pest	Lichens, mosses on bark: larvae feed on non-vascular plants attached to tree bark; no significant crop host; neutral to agricultural productivity.
37	*Lithostege griseata* (Denis & Schiffermüller, 1775)	Lepidoptera	*Geometridae*	Moderate pest	Isatis tinctoria (woad), crucifer weeds: a secondary pest of cruciferous crops and weed hosts; larvae feed on leaves and seed pods of Brassicaceae.
38	*Holcocera chalcofrontella* (Rebel, 1917)	Lepidoptera	*Gelechiidae*	Moderate pest	Stored plant material, dried bark: a minor stored-product gelechiid; larvae feed in dried organic debris; no significant field crop damage recorded.
39	*Macroglossum corythus* Walker, 1856	Lepidoptera	*Sphingidae*	Moderate pest	Rubiaceae (Ixora, Gardenia relatives), coffee relatives: an occasional garden and plantation pest in tropical and subtropical Asia; adults are pollinators.
40	*Mecyna flavalis* (Denis & Schiffermüller, 1775)	Lepidoptera	*Crambidae*	Moderate pest	Bindweed (Convolvulus arvensis), Calystegia spp.: field margin weed feeder; larvae restricted to Convolvulaceae; no direct crop damage documented.
41	*Tarache aperica* (Swinhoe, 1885)	Lepidoptera	*Noctuidae*	Moderate pest	Malvaceae, cotton (Gossypium spp.) relatives, hollyhock: minor foliage feeder; recorded from cotton-growing regions of Central and South Asia.
42	*Tinea columbariella* Wocke, 1877	Lepidoptera	*Tineidae*	Moderate pest	Feathers, dried organic matter, wool, stored grain debris: a keratin feeder in grain stores and poultry facilities; minor stored-product concern.
43	*Arhopalus rusticus* (Linnaeus, 1758)	Coleoptera	*Cerambycidae*	Moderate pest	Conifers, pine (Pinus spp.), spruce: larval galleries in weakened or fire-damaged wood; secondary pest following drought stress or fire damage in conifer forests.
44	*Protoschinia nuchalis* (Freyer, 1838)	Lepidoptera	*Noctuidae*	Moderate pest	Chenopodium spp., beet (Beta vulgaris) relatives: a minor noctuid pest in sugar beet-growing regions of Central Asia and Eastern Europe.
45	*Liguropia juniperi* (Reitter, 1895)	Coleoptera	*Cerambycidae*	Moderate pest	Juniper (Juniperus spp.): bark beetle causing larval galleries under bark; damages ornamental junipers and windbreak plantations in semi-arid zones.
46	*Anomala nimbosa* (Burmeister, 1844)	Coleoptera	*Scarabaeidae*	Moderate pest	Grass roots, turf, vegetable roots: a chafer grub causing localized root damage; adults defoliate low vegetation; minor but recurring garden and turf pest.
71	*Hybomitra sequax* (Williston, 1901)	Diptera	*Tabanidae*	Moderate pest	Female blood-feeder on livestock and large mammals; causes stress, blood loss, and reduced productivity in cattle, horses, and sheep; documented mechanical vector of animal pathogens (anthrax, anaplasmosis) in Central and South Asian pastoral systems.

77	*Platycleis grisea* (Fabricius, 1781)	Orthoptera	*Tettigoniidae*	Moderate pest	Grasses, cereal crops, wildflowers, and field margin vegetation: feeds on grass seeds, leaves, and flowers; recorded as a minor grazing pest in pasture and cereal field margins across the Palearctic steppe zone.
78	*Perissopygocoris pallidus* Linnavuori, 1962	Hemiptera	*Miridae*	Moderate pest	Phytophagous on herbaceous steppe plants and field margin vegetation across the Palearctic; pierces plant tissue and extracts sap; recorded from crop-adjacent habitats in Central Asia with potential for incidental crop feeding.
			BENEFICIAL INSECTS (23 species)		
47	*Chrysoperla carnea* (Stephens, 1836)	Neuroptera	*Chrysopidae*	Beneficial	Key aphid predator: larvae consume up to 200 aphids per week; also attacks mealybugs, mites, and small caterpillars; widely used in biological control programs globally.
48	*Harmonia axyridis* (Pallas, 1773)	Coleoptera	*Coccinellidae*	Beneficial	Predator of aphids, scale insects, and mites on crops; larvae and adults consume large numbers of pest insects; used in biocontrol but invasive in Europe and North America.
49	*Badister bullatus* (Schrank, 1798)	Coleoptera	*Carabidae*	Beneficial	Ground predator of small insects and soil-dwelling arthropods; nocturnal; inhabits moist crop margins and grassland; contributes to natural suppression of soil pests.
50	*Calosoma auropunctatum* (Herbst, 1784)	Coleoptera	*Carabidae*	Beneficial	Active predator of cutworms (Agrotis spp.) and armyworms (Spodoptera spp.); a key biocontrol carabid in steppe agroecosystems of Central Asia and Eastern Europe.
51	*Chlaenius velutinus* (Duftschmid, 1812)	Coleoptera	*Carabidae*	Beneficial	Carabid predator of small insects, slugs, and soil arthropods in moist crop margins and irrigated fields; contributes to suppression of soil pest populations.
52	*Didea intermedia* Loew, 1854	Diptera	*Syrphidae*	Beneficial	Hoverfly: adults pollinate flowers; larvae are active predators of aphid colonies; recognized as a beneficial insect in integrated pest management.
53	*Badister neopulchellus* Liebke, 1929	Coleoptera	*Carabidae*	Beneficial	Carabid ground predator in grassland and cereal field margins; feeds on invertebrate eggs and small arthropods; contributes to natural pest regulation.
54	*Ichneumon laetus* Gravenhorst, 1829	Hymenoptera	*Ichneumonidae*	Beneficial	Endoparasitoid wasp: oviposits inside lepidopteran larvae (Noctuidae, Pyralidae); kills host larvae during development; important in biological control of caterpillar pests.
55	*Labidura riparia* (Pallas, 1773)	Dermaptera	*Labiduridae*	Beneficial	Omnivorous predator of small insects, eggs, and larvae in rice paddies and irrigated crops; voracious predator of fall armyworm eggs and small caterpillars.
56	*Notonecta glauca* Linnaeus, 1758	Hemiptera	*Notonectidae*	Beneficial	Aquatic predator: backswimmer that controls mosquito larvae, chironomid larvae, and other aquatic insects in rice paddies and irrigation channels.
57	*Myrmeleon obscurus* Rambur, 1842	Neuroptera	*Myrmeleontidae*	Beneficial	Antlion: larvae construct pit traps in dry soil and consume ants, beetles, and crawling insects; adults also predatory; reduces surface-dwelling insect populations.
58	*Ophion luteus* (Linnaeus, 1758)	Hymenoptera	*Ichneumonidae*	Beneficial	Ectoparasitoid of noctuid moth larvae including cutworms and armyworms; lays eggs on host externally; a key natural regulation agent of caterpillar pest populations.
59	*Poecilus puncticollis* (Dejean, 1828)	Coleoptera	*Carabidae*	Beneficial	Carabid predator of aphids, caterpillars, and soil-dwelling arthropods in cereal fields; nocturnal; important component of biocontrol in steppe agroecosystems.
60	*Scarites subterraneus* Fabricius, 1775	Coleoptera	*Carabidae*	Beneficial	Burrowing ground beetle: actively pursues and kills cutworm larvae in vegetable and row crop soils; one of the most effective carabid predators of underground pests.
61	*Stethoconus pyri* (Josifov, 1971)	Hemiptera	*Miridae*	Beneficial	Predatory mirid bug: feeds specifically on lace bugs (Tingidae) and spider mites on pear (Pyrus spp.) and apple (Malus spp.); an important natural enemy in orchards.
62	*Quedius capucinus* (Gravenhorst, 1802)	Coleoptera	*Staphylinidae*	Beneficial	Rove beetle: predator of fly larvae, mites, and small invertebrates in moist crop debris and soil; contributes to decomposer food web regulation.
63	*Ectomocoris ululans* (Rossi, 1790)	Hemiptera	*Reduviidae*	Beneficial	Assassin bug: attacks caterpillars, small beetles, and other crop insects; recorded as a beneficial predator in rice, vegetable, and tropical plantation crops.
64	*Plistospilota guineensis* Giglio-Tos, 1915	Mantodea	*Mantidae*	Beneficial	Predatory mantid: ambush predator feeding on small insects, caterpillars, and eggs in tropical and subtropical crops; general predator with no crop damage recorded.
65	*Stenopoda spinulosa* Reuter, 1882	Hemiptera	*Reduviidae*	Beneficial	Assassin bug: ambush predator of moths, beetles, and other large insects; recorded from semi-arid shrubland and crop margins; no phytophagous role known.
66	*Calliphora nigribarbis* Vollenhoven, 1863	Diptera	*Calliphoridae*	Beneficial	Blowfly: adults are incidental pollinators; larvae decompose carrion and organic matter contributing to nutrient cycling; no crop pest role recorded.
74	*Laccophilus quadrilineatus* Schaum, 1864	Coleoptera	*Dytiscidae*	Beneficial	Aquatic predatory diving beetle: larvae and adults actively hunt mosquito larvae, chironomid larvae, and other small aquatic invertebrates in irrigation channels, rice paddies, and field ponds; contributes to biological suppression of aquatic pest insects.

80	*Orthotylus marginalis* Reuter, 1883	Hemiptera	*Miridae*	Beneficial	Omnivorous predatory mirid on alder (Alnus spp.), willow (Salix spp.), apple (Malus spp.), and currant (Ribes spp.); adults actively prey on aphids (Aphididae) and spider mites (Tetranychidae); recognized as a natural enemy in orchard and fruit crop systems across the Palearctic.
82	*Zavrelimyia barbatipes* (Kieffer, 1911)	Diptera	*Chironomidae*	Beneficial	Predatory non-biting midge: aquatic larvae are active predators of chironomid eggs, small invertebrate larvae, and oligochaetes in riverine and irrigation channel sediments; contributes to biological suppression of aquatic pest midge populations.
83	*Rhantus elisabethae* Régimbart, 1895	Coleoptera	*Dytiscidae*	Beneficial	Predatory aquatic diving beetle: larvae and adults consume mosquito larvae, aquatic fly larvae, and other invertebrates in ponds, ditches, and irrigation channels; a beneficial predator in rice-paddy and irrigated crop systems.
			NEUTRAL (10 species)		
67	*Tasiagma ciliata* Neboiss, 1977	Trichoptera	*Tasimiidae*	Neutral	Aquatic caddisfly; larvae inhabit fast-flowing rainforest streams in southeastern Australia (Salix and Eucalyptus litter); no crop or agricultural association. [Order corrected to Trichoptera:Tasimiidae per ITIS and Hughes et al. 1998].
68	*Amphinemura linda* (Claassen, 1936)	Plecoptera	*Nemouridae*	Neutral	Aquatic stonefly; larvae develop in cool, clean freshwater streams; adults do not feed; no direct agricultural significance. [Treated as junior synonym of A. palmeni per Boumans & Baumann 2012, Zootaxa 3537].
69	*Dixella martinii* (Lackschewitz, 1925)	Diptera	*Dixidae*	Neutral	Meniscus midge; larvae live in the surface film of slow-moving wetland water; adults non-feeding; aquatic larvae feed on algal film; no crop association or economic significance.
70	*Dicranota subtilis* Loew, 1871	Diptera	*Pediciidae*	Neutral	Hairy-eyed crane fly; larvae develop in waterlogged soil and stream margins feeding on decomposing vegetation; no crop damage recorded.
72	*Dolichopeza albipes* (Ström, 1768)	Diptera	*Tipulidae*	Neutral	Large crane fly; larvae develop in waterlogged moss and wet woodland litter feeding on decaying organic matter; no crop damage role; decomposer only.
73	*Eupterycyba jucunda* (Herrich-Schäffer, 1837)	Hemiptera	*Cicadellidae*	Neutral	Leafhopper restricted to birch (Betula spp.); no crop host; no significant economic damage to cultivated species documented in literature.
75	*Odontotaenius disjunctus* (Illiger, 1800)	Coleoptera	*Passalidae*	Neutral	Bess beetle; obligate decomposer of decaying hardwood logs; subsocial with parent-offspring cooperation; no crop pest role recorded.
76	*Ephemera vulgata* Linnaeus, 1758	Ephemeroptera	*Ephemeridae*	Neutral	Mayfly; aquatic nymphs burrow in silt of rivers and lakes feeding on diatoms and fine organic particles; short-lived winged adults do not feed; no crop significance.
79	*Pinacoplus didymogramma* (Bremer, 1864)	Lepidoptera	*Noctuidae*	Neutral	Owlet moth (Noctuidae: Metoponiinae); sole species of genus Pinacoplus; larval biology and host plant undescribed in published literature; no confirmed crop damage records. [Order corrected to Lepidoptera:Noctuidae per GBIF; previously misassigned to Hemiptera:Miridae].
81	*Notoxus cavifrons* LeConte, 1851	Coleoptera	*Anthicidae*	Neutral	Antlike flower beetle; found in grain stores, sandy soil, and crop debris; larvae likely predatory or detritivorous; no confirmed crop damage role; neutral in agricultural settings.

Summary Statistics: Total species: 83 | High-damage pests: 17 | Moderate pests: 31 | Beneficial insects: 23 | Neutral: 10 (reclassified from Neutral/data-deficient; confirmed non-pest non-beneficial)
