# Supplementary material for: Habitat-Driven Insect Community Structure in an Arid Agroecosystem of Xinjiang, China: Integrated Light-Trap Monitoring and DNA Barcoding Evidence
Source: Insects. 2026 Jul 28;17(8):777. doi: 10.3390/insects17080777 (PMC13513039; doi:10.3390/insects17080777)
Supplement: Supplementary file 1 [file insects-17-00777-s001.zip › Supplementary File S2.pdf]

Light-trap monitoring across three habitat types: xerophytic shrubland (L1), agro-horticultural garden (L2), riparian corridor (L3)

Table S2 Beneficial insect families recorded at study sites (n = 10)

No.	Family	Representative taxa	Ecological role	Notes	Locations	Importance
**1**	*Apidae*	*Apis mellifera, Bombus spp.*	Pollination	Primary pollinators of fruit trees, vegetables and wild flora; essential for crop reproduction at Location 2 (agro-horticultural site).	1, 2, 3	**High**
**2**	*Coccinellidae*	*Coccinella spp., Harmonia spp.*	Aphid predation	Predators of aphids, scale insects and mites; recorded at highest abundance at Location 1 (7 genera). Reduce dependence on chemical pesticides.	1, 2, 3	**High**
**3**	*Chrysopidae*	*Chrysoperla carnea*	Soft-bodied pest predation	Lacewing larvae consume aphids, whitefly and thrips at all three sites; adults are important pollinators of small-flowered plants.	1, 2, 3	**Moderate**
**4**	*Carabidae*	*Carabus spp., Cicindela spp.*	Ground-dwelling predation	Highest family diversity in the study (10 genera, Location 1). Prey on soil pests, weed seeds and small invertebrates; widely used as habitat quality bioindicators.	1, 2, 3	**High**
**5**	*Syrphidae*	*Episyrphus spp., Syrphus spp.*	Pollination / aphid predation	Adults pollinate flowers; larvae are specialist aphid predators. Recorded at Locations 2–3 near abundant flowering vegetation.	2, 3	**Moderate**
**6**	*Vespidae*	*Polistes spp., Vespa spp.*	Caterpillar predation	Social wasps provision nests with caterpillars and soft insects, substantially reducing herbivorous pest loads across all sites.	1, 2, 3	**Moderate**
**7**	*Myrmeleontidae*	*Myrmeleon spp.*	Pit-trap predation (larval)	Most species-rich family recorded (22 genera, Location 1). Antlion larvae regulate ant and small arthropod populations in sandy, arid soils.	1, 2, 3	**High**
**8**	*Gomphidae*	*Gomphus spp., Ophiogomphus spp.*	Aquatic predation / indicator	Larvae control mosquito and chironomid populations in the riparian corridor (Location 3); adults prey on aerial insects. Sensitive bioindicators of water quality.	1, 2, 3	**Moderate**
**9**	*Libellulidae*	*Libellula spp., Sympetrum spp.*	Aerial mosquito predation	Highly mobile predators consuming mosquitoes, gnats and small flies; most abundant near the water stream at Location 3.	1, 2, 3	**Moderate**
**10**	*Staphylinidae*	*Staphylinus spp., Ocypus spp.*	Decomposition / soil health	Rove beetles decompose organic matter and prey on fly larvae in the soil layer, improving nutrient cycling and soil aeration at Locations 1 and 3.	1, 3	**Moderate**

Importance assessed on the basis of species richness, abundance, functional role, and relevance to ecosystem service provision. High = documented quantitative service; Moderate = supporting role with indirect benefits.

Table S3 Harmful and pest insect families recorded at study sites (n = 10)

No.	Family	Representative taxa	Damage type	Notes	Locations	Risk level
**1**	*Acrididae*	*Locusta migratoria, Schistocerca spp.*	Defoliation of crops and grasslands	Grasshoppers and locusts recorded at all sites (4 genera, Locations 1 and 3). Swarm events cause rapid and extensive removal of vegetative cover.	1, 2, 3	**High**
**2**	*Noctuidae*	*Helicoverpa zea*	Borer / polyphagous feeder	Corn earworm ranked 3rd in total abundance (5,209 specimens). Attacks cotton bolls, maize ears and tomato fruit; contributes substantially to yield loss.	1, 2, 3	**High**
**3**	*Scarabaeidae*	*Oryctes rhinoceros*	Structural boring (trunk and roots)	Rhinoceros beetle ranked 4th in total abundance (5,206 specimens). Adults bore into palm and tree trunks; larvae destroy root systems over multi-year periods.	1, 2, 3	**High**
**4**	*Crambidae*	*Chilo suppressalis, Ostrinia spp.*	Stem boring of cereals	High genus richness at Location 2 (8 genera, 9 species). Larvae bore into stems of maize, rice and sorghum, causing lodging and partial crop loss.	1, 2, 3	**High**
**5**	*Erebidae*	*Spodoptera litura, Arctia spp.*	Defoliation (polyphagous)	Armyworms and tiger moths recorded across all sites. Larvae consume foliage of vegetables, cotton and ornamentals; periodic outbreaks cause widespread damage.	1, 2, 3	**High**
**6**	*Geometridae*	*Biston betularia, Eupithecia spp.*	Defoliation of woody plants	Geometrid caterpillars (inchworms) feed on tree and shrub foliage; highest diversity at Location 3 (7 genera and species). Repeated outbreaks weaken host trees.	1, 2, 3	**Moderate**
**7**	*Cicadellidae*	*Empoasca spp., Nephotettix spp.*	Sap-sucking / virus transmission	Leafhoppers inject toxic saliva, causing stippling and necrosis, and vector Maize streak virus and other phytopathogens across all three study sites.	1, 2, 3	**High**
**8**	*Pentatomidae*	*Nezara viridula, Halyomorpha halys*	Seed and fruit sap-feeding	Stink bugs feed on seeds and fruit, injecting phytotoxic saliva causing cosmetic and yield damage. Present at all sites with up to 3 genera/species.	1, 2, 3	**Moderate**
**9**	*Cossidae*	*Cossus cossus, Zeuzera pyrina*	Woodboring (trunk and branch)	Goat moth and leopard moth larvae bore through living wood for 2–4 years, causing structural weakening and mortality of fruit and ornamental trees.	1, 2, 3	**Moderate**

**10**	*Dytiscidae*	*Dytiscus marginalis*	Predation on aquatic fauna	Predatory diving beetles attack fish fry and amphibian larvae in irrigation channels and ponds adjacent to Location 3 (riparian corridor).	1, 2, 3	**Low–Moderate**

Risk level: High = direct crop/structural economic damage with outbreak potential; Moderate = localised or secondary damage; Low–Moderate = conditional pest status dependent on population density and host availability.

Table S4 Top 15 ecologically important insect families — ranked by functional significance

Rank	Family	Primary function	Ecological significance	Priority
1	*Myrmeleontidae*	Trophic regulation	Most species-rich family recorded (22 genera, Location 1). Antlion larvae are specialist predators structuring invertebrate assemblages in arid soils. High abundance confirms functional importance across all sites.	**Critical**
2	*Carabidae*	Keystone predation / bioindicator	Ten genera recorded at Location 1 — highest within-family richness among predatory families. Ground beetles link soil and canopy food webs and are among the most validated indicators of habitat disturbance in global literature.	**Critical**
3	*Apidae*	Pollination ecosystem service	Bee pollination underpins reproductive success of all entomophilous plant species at the garden site. Loss of this service would cascade to reduced fruit set, reduced bird food availability and ultimately lower plant diversity.	**Critical**
4	*Coccinellidae*	Biological pest control	Seven genera at Location 1; adults consume 200–500 aphids day^{-1}. Presence across all sites indicates a stable prey–predator equilibrium and reduces agrochemical requirements at the agro-horticultural site.	**High**
5	*Rhyacophilidae*	Aquatic ecosystem health indicator	Free-living caddisfly larvae are sensitive to organic pollution and thermal stress. Seven to nine species recorded across all sites; their sustained presence signals acceptable water quality in the Kekedala riparian corridor.	**High**
6	*Crambidae*	Herbivory / nutrient cycling	Eight genera at Location 2 make this the most diverse herbivore family at the garden site. As primary consumers, larvae transfer plant biomass to higher trophic levels; frass deposition accelerates soil nitrogen cycling.	**High**
7	*Erebidae*	Trophic transfer / decomposition	Outbreak biomass generates nutrient pulses via frass and cadavers. Adult moths are important prey for insectivorous bats and birds, connecting the insect and vertebrate components of the regional food web.	**High**
8	*Geometridae*	Herbivory / avian food subsidy	Seven genera at Location 3. Geometrid caterpillars are a primary dietary item for passerine birds during the breeding season, indirectly supporting avian diversity in urban and peri-urban landscapes.	**High**
9	*Gomphidae / Libellulidae*	Aquatic–terrestrial linkage	Odonate larvae connect aquatic and terrestrial ecosystems: larvae regulate dipteran larvae in water, while adult mortality transfers aquatic-derived nutrients to terrestrial habitats. Key indicators of riparian corridor health.	**High**
10	*Vespidae*	Prey population regulation	Social wasps remove caterpillars and flies across all three sites, providing measurable biological control. Nest construction also facilitates wood decomposition and microhabitat creation for secondary cavity-nesting species.	**Moderate–High**
11	*Acrididae*	Grassland energy flow	Four genera at Locations 1 and 3. Grasshoppers convert plant biomass to animal protein at high efficiency, supporting predatory insects, insectivorous birds and lizards — particularly important in the xerophytic shrubland habitat.	**Moderate–High**

**12**	*Chrysopidae*	Integrated pest regulation	Generalist predators recorded across all three sites. Lacewing presence indicates sufficient prey diversity and structural complexity; their consistent occurrence supports natural regulation without site-specific specialisation.	**Moderate**
**13**	*Scarabaeidae*	Decomposition / soil engineering	Four genera at each site. Dung and carrion beetles bury organic matter and improve soil porosity and fertility through tunnelling. Among the most functionally important decomposer groups in semi-arid ecosystems.	**Moderate**
**14**	*Staphylinidae*	Soil invertebrate regulation	Mobile generalist predators in the soil and litter layer. Regulation of fly larvae, nematodes and soil micro-arthropods by rove beetles contributes to below-ground food web stability at Locations 1 and 3.	**Moderate**
**15**	*Cicadidae*	Soil aeration / nutrient pulse	Nymphs aerate soils during multi-year subterranean development; mass adult emergences deposit substantial nutrient pulses and serve as critical food resources for vertebrate predators, amplifying productivity across trophic levels.	**Moderate**

Priority ranking integrates: (1) trophic position and functional uniqueness; (2) relative abundance in present survey; (3) sensitivity to environmental change (bioindicator value); (4) breadth of ecosystem services delivered. Critical = irreplaceable function with documented cascade effects; High = substantial trophic or service role; Moderate = supporting role across multiple functions.
